# Dysregulated glucose metabolism in the visual cortex of human subjects with mild cognitive impairment and Alzheimer’s disease

**DOI:** 10.3389/fnagi.2026.1710075

**Published:** 2026-04-09

**Authors:** Ulrik N. Mjaaseth, Ming-Fo Hsu, Joseph Arballo, Fawaz G. Haj, Viharkumar Patel

**Affiliations:** 1 Department of Nutrition, University of California, Davis, Davis, CA, United States; 2 Comprehensive Cancer Center, University of California, Davis, Sacramento, CA, United States; 3 Division of Endocrinology, Diabetes, and Metabolism, Department of Internal Medicine, University of California, Davis, Sacramento, CA, United States; 4 Department of Pathology, University of California, Davis, Sacramento, CA, United States

**Keywords:** Alzheimer’s disease, Braak staging, glucose metabolism, insulin signaling, mild cognitive impairment, mitochondrial function, neurodegeneration, visual cortex

## Abstract

**Introduction:**

Alzheimer’s disease (AD) is characterized by progressive neurodegeneration and impaired glucose metabolism. While most studies focus on heavily affected brain regions such as the hippocampus and prefrontal cortex, the visual cortex remains relatively preserved in early AD and provides an opportunity to examine metabolic alterations that precede widespread pathology.

**Methods:**

Postmortem human visual cortex samples were obtained from control, mild cognitive impairment (MCI), and AD subjects without non-AD neuropathologic conditions. Untargeted metabolomics was performed using liquid chromatography–mass spectrometry, and expression of key metabolic, inflammatory, and AD-related genes was measured by quantitative PCR. Data analysis was conducted using MetaboAnalyst and R.

**Results:**

Metabolomic profiling revealed progressive disruptions in glucose metabolism, and mitochondrial function across MCI and AD subjects. Gene expression analyses showed reduced levels of glycolytic enzymes (*HK1*, *PFKM*, *PKM1*), mitochondrial regulators (*PDHA1*, *NDUFC1*), and the neuronal glucose transporter *SLC2A3*. Insulin signaling was altered, with decreased *IDE* and increased *INSR* and *PTPN1* gene expression. Inflammatory markers including *TNF*, *IL1B*, and *GFAP* were elevated in AD. Sex-stratified analyses revealed both shared and distinct metabolic signatures, particularly within glucose and mitochondrial pathways. Several metabolic gene changes correlated negatively with Braak stage, highlighting a progressive decline in energy metabolism alongside tau pathology.

**Discussion:**

These findings demonstrate early and progressive metabolic dysfunction in the visual cortex of MCI and AD subjects. Even in a region with limited structural pathology, profound alterations in energy metabolism were observed, underscoring its central role in AD pathogenesis and highlighting improving neuronal metabolic function as a promising target for therapeutic intervention.

## 1 Background

Alzheimer’s disease (AD) is the most widespread form of dementia, affecting approximately 1 in 3 people over the age of 85 in the United States (Hebert et al., 2013). As of 2020, an estimated 58 million people worldwide had dementia (with AD representing the majority), a number expected to balloon to 152 million by 2050 (Baviskar and Mahajan, 2026). The rising prevalence and exorbitant costs of treating AD underscore the urgent need for a more comprehensive understanding of disease pathogenesis and the development of novel therapeutics.

AD is characterized by a steady decline in cognitive function, accompanied by neuron death and brain atrophy. Historically, the neurodegeneration observed in AD was thought to be driven by the accumulation of amyloid beta (Aβ) plaques and tau neurofibrillary tangles (Kent et al., 2020). However, this has been expanded upon, as Aβ plaque frequency does not closely correlate with the severity of cognitive decline, and as the complexity and multifactorial nature of AD pathogenesis have come to be appreciated (Bowirrat et al., 2025).

Lifestyle factors, including exercise, good nutrition, and maintaining a healthy weight, are all associated with lower dementia risk (Kazibwe et al., 2024; Gomez-Pinilla, 2008). These same factors also support metabolic fitness, defined as the body’s capacity to efficiently process and utilize energy from nutrients through various metabolic pathways (Qureshi et al., 2024; Craft, 2009). The putative protective effects of improved metabolic fitness against AD are likely multifaceted. Among these, improved glucose control is particularly neuroprotective (Mosconi, 2005; Zhang et al., 2023). This is not surprising given the brain’s unparalleled requirement for glucose (Mergenthaler et al., 2013). Indeed, multiple fluorodeoxyglucose positron emission tomography studies have shown that subjects with MCI or AD consistently have reduced brain glucose metabolic rates compared to cognitively normal controls (Friedland et al., 1985; Herholz, 2003; Chen et al., 2021). Previously, it was believed that altered glucose metabolism in AD patients was a side effect of brain atrophy (Ibáñez et al., 1998). However, newer findings reveal that altered cerebral glucose metabolism occurs independently of atrophy, is present in early AD, and appears before dementia symptoms first manifest (Zhang et al., 2023; Wang et al., 2022). Further corroborating this, individuals carrying the apolipoprotein E epsilon 4 allele (APOE ε4) exhibit reductions in glucose utilization in a manner similar to AD-diagnosed subjects before dementia symptoms manifest (Vanya et al., 2025). Importantly, a central component of metabolic dysfunction in AD is the loss of the brain’s metabolic flexibility, which contributes to disease pathogenesis (Bajinka et al., 2026).

Insulin is a key regulator of whole-body glucose homeostasis (Saltiel and Kahn, 2001). It crosses the blood–brain barrier and plays a crucial role in neuronal health (Milstein and Ferris, 2021). Insulin resistance and type 2 diabetes (T2D) are associated with AD, with one study reporting that approximately 81% of AD patients had either T2D or impaired fasting glucose (Janson et al., 2004). Multiple studies demonstrate that AD patients exhibit elevated neuronal insulin resistance and reduced expression of the insulin receptor and insulin like-growth factor receptors required for insulin signaling (Rivera et al., 2005; Talbot et al., 2012; Steen et al., 2005). These findings led some researchers to consider AD to be a “type 3 diabetes” (Steen et al., 2005; de la Monte and Wands, 2008). At the molecular level, disruption of glucose metabolism is partly explained by reduced expression in glucose transporters (GLUTs) in AD brains (Kyrtata et al., 2021). Metabolomic studies have further identified alterations in glucose metabolism in the brains of AD subjects (Patel et al., 2024). Additionally, mitochondrial dysfunction and reduced ATP-generating capacity are established features of AD (Ashleigh et al., 2023; Bhatia et al., 2022), collectively indicating impairments in energy metabolism at multiple, interconnected levels.

AD typically manifests initially in the hippocampus and entorhinal cortex, brain regions crucial for memory formation. As the disease progresses, other cortical areas are affected. Due to this well-established pathology progression, most postmortem studies of AD brains focus on the hippocampus or the frontal or temporal lobes (Xu et al., 2019; DeTure and Dickson, 2019). To further probe metabolic alterations in the AD brain, we henceforth have performed an untargeted metabolomics analysis and assessed the gene expression of key metabolic enzymes in postmortem visual cortex tissues of subjects with no cognitive decline (control), mild cognitive impairment (MCI), or confirmed AD diagnosis. By investigating the visual cortex, we aim to reveal nuanced metabolic alterations. The visual cortex has less accumulation of Aβ plaques, atrophy, and inflammation (Arnold et al., 1991; La Joie et al., 2012). Accordingly, we postulate that the visual cortex may imperfectly “model” earlier stages of AD, and the findings may reflect early stages of AD etiology. This relative preservation allows metabolic disturbances to be examined in a region less confounded by extensive neurodegeneration, thereby highlighting early bioenergetic vulnerabilities that may be relevant for therapeutic intervention.

## 2 Methods

### 2.1 Subject information

Postmortem visual cortex brain tissue was collected and frozen from donors with a clinical diagnosis of sporadic AD, MCI, or control, and acquired from the Banner Sun Health Research Institute (AZ, USA). Brain tissue was stored at −80 °C. Postmortem diagnosis of AD was established through neuropathologic examination performed at Banner Sun Health Research Institute. An exclusion criterion for these subjects was a non-AD neuropathologic conditions, e.g., Parkinson’s disease, dementia with Lewy bodies, progressive supranuclear palsy, etc., and significant non-neurodegenerative diseases, such as infarct, hemorrhage, infection, etc. All subjects were matched based on age, sex, and postmortem interval. Postmortem demographic information for the subjects is listed in Table 1, with details for each subject provided in Supplementary Table 1. All experiments involving human-derived materials were conducted in accordance with institutional guidelines and approved protocols at the University of California, Davis. Appropriate ethical approval was obtained, and all procedures complied with relevant regulations for research.

**Table 1:** Demographic characteristics and apolipoprotein E (APOE) genotype of subjects.

	**Clinical Diagnosis**		
**Characterstics**	**Control, No Cognitive Decline**	**Mild Cognitive Impairment**	**Alzheimer's Disease**
Population	20	20	20
Male	10	7	11
Female	10	13	9
Race	Caucasian	Caucasian	Caucasian
Age Range (yrs)	53- ≥ 90	77- ≥ 90	73- ≥ 90
Braak Stage	I-IV	III-VI	V-VI
Postmortem Interval (hrs)	3.44 ± 1.13	3.65 ± 1.55	3.42 ± 0.62
APOE Genotype			
ε2/ε3	5	4	1
ε2/ε4	0	0	1
ε3/ε3	12	11	7
ε3/ε4	3	5	9
ε4/ε4	0	0	2

### 2.2 Metabolomic analysis

Visual cortex tissue samples were sent to Metabolon (NC, USA) for untargeted metabolomic analysis, as previously reported (Patel et al., 2024). Upon receipt, samples were accessioned into a Laboratory Information Management System, stored at −80 °C, and randomly processed to minimize batch effects. Proteins were precipitated with methanol, and the resulting supernatants were divided into four fractions. These individual fractions were run in analysis for one of the following methods: Two fractions were run on the reversed-phase (RP) ultra-high-performance liquid chromatography–tandem mass spectrometry (UPLC-MS/MS) method with positive ion mode electrospray ionization (ESI), one fraction was for analysis by RP/UPLC-MS/MS with negative ion mode ESI, and one for analysis by hydrophilic interaction liquid chromatography UPLC-MS/MS with negative ion mode ESI. This multi-platform approach ensured comprehensive coverage of the metabolome across lipid, amino acid, carbohydrate, nucleotide, and energy metabolism pathways. Raw spectral data were processed using Metabolon’s proprietary informatics pipeline, which performs peak detection, metabolite identification, and normalization based on a library of >5,400 authenticated standards and 7,000 structurally uncharacterized recurring biochemicals.

Rigorous quality control procedures were employed, including pooled technical replicates, process blanks, and internal standards to monitor extraction efficiency and instrument performance. Peak areas were used for relative quantification, and data normalization was applied to correct for day-to-day analytical variation. Named metabolites are confirmed through multiple independent measurements, such as accurate mass, retention time, and MS/MS spectra, and are matched to authentic standards (level 1 identification). An asterisk after a biochemical name indicates that the compound has not been confirmed using a reference standard, but its identity is still considered highly reliable (level 2 identification) (Schymanski et al., 2014).

Raw peak values were log-transformed and median-scaled for normalization before uploading data into MetaboAnalyst 6.0 (Pang et al., 2024), and no further normalization was performed. Data were checked for integrity and filtered by interquartile range. Specific statistical analyses are described in the figure legends.

### 2.3 RNA extraction and quantification

Frozen tissue from the visual cortex of each donor was homogenized to extract total RNA using TRIzol reagent (Invitrogen). The RNA quantity and quality were assessed using a NanoDrop One spectrophotometer (Thermo Fisher Scientific). Subsequently, 2 μg total RNA was used to generate cDNA, which was synthesized using a high-capacity cDNA reverse transcription kit (Applied Biosystems). The gene expression level was assessed using SsoAdvanced Universal SYBR Green Supermix (Bio-Rad) and relevant primer pairs. Threshold cycle (Ct) values were determined using CFX Duet Real-Time PCR System (Bio-Rad). For normalization of gene expression, ubiquitin-conjugating enzyme E2 D2 (*UBE2D2*) was used as the housekeeping gene for these human samples as recommended by Rydbirk et al. (2016) and Leduc et al. (2011). Each gene of interest was analyzed in duplicate on a 96-well plate, with its corresponding housekeeping gene included on the same plate for normalization. Relative mRNA expression levels were quantified using the 2ˆ(−delta delta Ct) method. The sequences of the primers used are listed in Supplementary Table 2.

### 2.4 Statistical analyses and data visualization

Gene expression was analyzed using Rstudio version 2023.06.2. Statistical tests employed are described in figure legends. ggplot2, MetaboAnalyst 6.0 and Biovenn were utilized to generate graphics (Pang et al., 2024; Hulsen et al., 2008).

## 3 Results

### 3.1 Metabolite profiles were altered in the visual cortex of the MCI and AD subjects compared to controls

**Figure 1:**
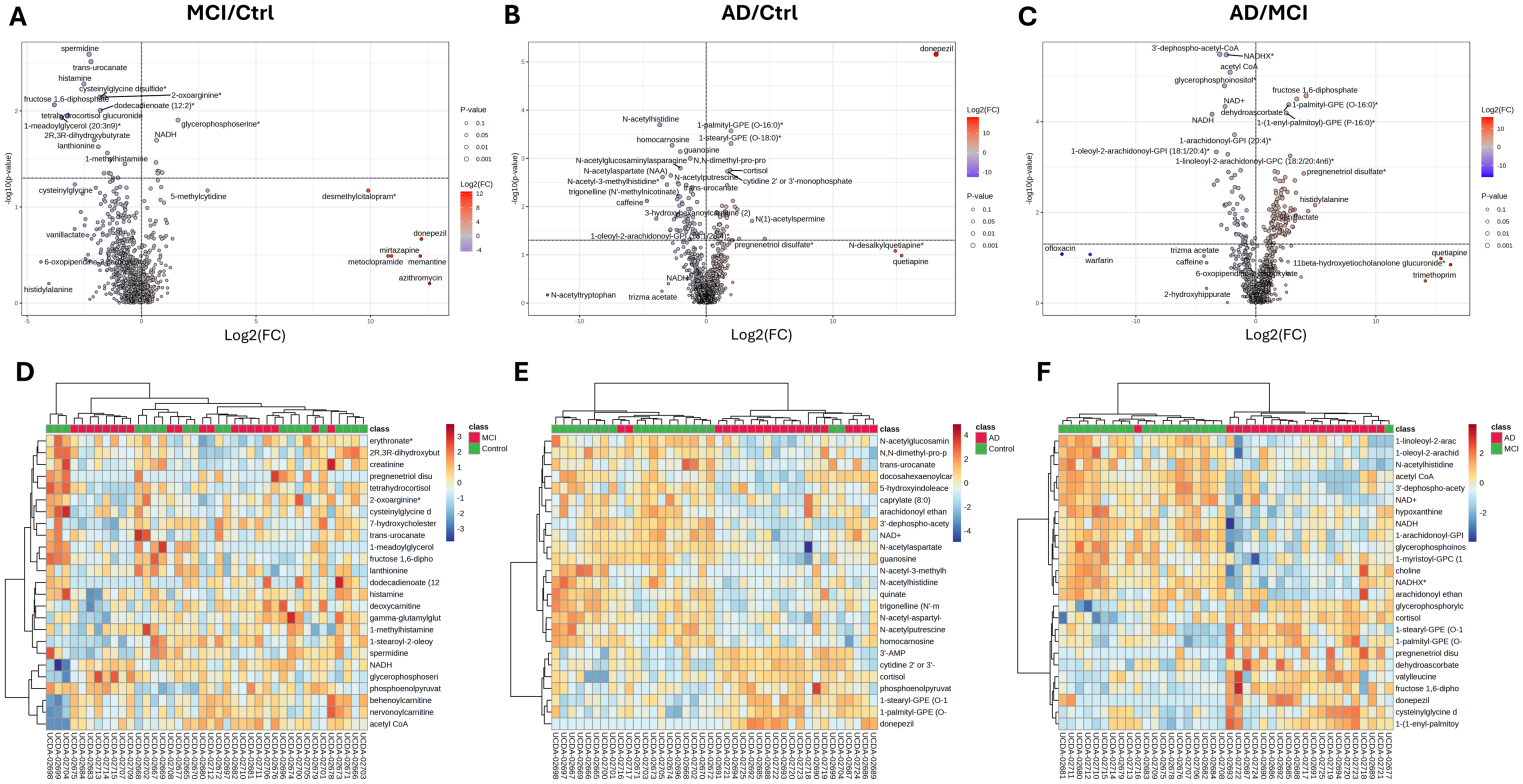
Metabolite profile was altered in the visual cortex of the AD group. Volcano plots show the differential metabolite levels in comparisons of **(A)** MCI/control (Ctrl), **(B)** AD/Ctrl, and **(C)** AD/MCI. The −log10(*p*-value) from two-group *t*-tests is plotted on the *Y*-axis, while the log2 fold change (Log2(FC)) is shown on the *X*-axis. Metabolites shown above horizontal dashed line are statistically significant *p* < 0.05. Metabolites colored blue are decreased, while those in red are increased. Hierarchical clustering and heatmap of top 25 most significant metabolites by *p* value in **(D)** MCI/Ctrl, **(E)** AD/Ctrl, and **(F)** AD/MCI. Red and blue indicate higher and lower metabolite abundance, respectively. The similarity measurement for clustering was done on normalized data with Euclidean distance measurements and the Ward clustering method. Statistical significance of metabolite ratios was determined using two-group *t*-tests *p* < 0.05.

To investigate metabolic dysregulation in AD, postmortem visual cortex samples from AD, MCI and control (Ctrl) donors were subjected to untargeted metabolomics, which identified 921 metabolites. Two-group t-tests revealed 26 statistically significant altered metabolites in MCI vs. Ctrl (Figure 1A), 108 in AD vs. Ctrl (Figure 1B), and 173 in AD vs. MCI (Figure 1C). Lists of all significant metabolites for the different comparisons MCI vs Ctrl are in supplementary data sheet 1, AD vs Ctrl are in supplementary data sheet 2, and AD vs MCI are in supplementary data sheet 3.

Hierarchical clustering of the top 25 most significantly altered metabolites showed no distinct clustering between MCI and Ctrl subjects (Figure 1D), suggesting that metabolic alterations in MCI remain subtle. In contrast, distinct clustering was observed in AD vs. Ctrl (Figure 1E) and AD vs. MCI (Figure 1F), indicating a more pronounced metabolic shift with disease progression. These findings suggest that metabolic alterations in the visual cortex in MCI subjects may be limited in scope, whereas AD is marked by a distinct metabolic profile consistent with progressive pathophysiological changes.

### 3.2 Energy-related pathways were significantly altered in the visual cortex of both MCI and AD subjects

**Figure 2:**
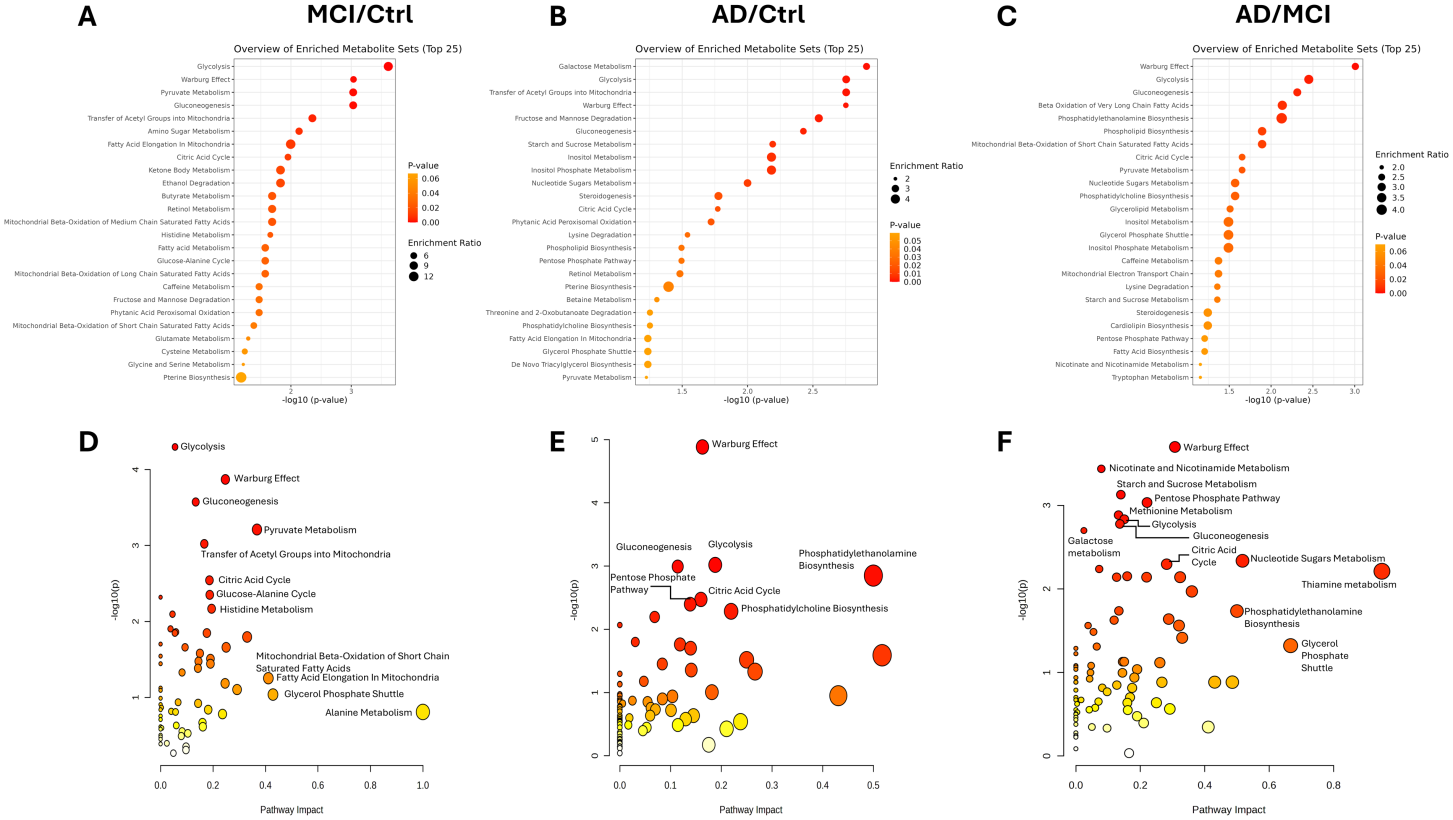
Enrichment and pathway analysis reveals alterations in energy generating processes in the visual cortex of MCI and AD groups. Metabolite set enrichment analysis using the Small Molecule Pathway Database (SMPDB) reveals alterations in energy metabolism in MCI and AD groups. Bubble plots are used to illustrate level of enrichment with bubble size corresponding to enrichment ratio and color denoting statistical significance. Bubble plots show comparisons for **(A)** MCI/Ctrl, **(B)** AD/Ctrl, and **(C)** AD/MCI. Scatter plots visualize pathway enrichment using SMPDB for **(D)** MCI/Ctrl, **(E)** AD/Ctrl, and **(F)** AD/MCI. Statistical significance of metabolite ratios was determined using two-group *t*-tests *p* < 0.05.

To elucidate metabolic pathway dysregulation in MCI and AD, significantly altered metabolites were subjected to pathway enrichment analysis using the Small Molecule Pathway Database (SMPDB) (Jewison et al., 2013) and Kyoto Encyclopedia of Genes and Genomes (KEGG) (Kanehisa et al., 2025). In the MCI vs. Ctrl comparison using SMPDB: Glycolysis, Warburg Effect, Pyruvate Metabolism, Gluconeogenesis, and the Transfer of Acetyl Groups into the mitochondria emerged as the most enriched pathways (Figure 2A). The functional overlap among these pathways suggests a coordinated disruption of central glucose metabolism during disease progression. Notably, KEGG enrichment analysis corroborated these findings, identifying Glycolysis/Gluconeogenesis, Pyruvate Metabolism, and the tricarboxylic acid (TCA) cycle as the top-enriched pathways (Supplementary Figure 1).

In AD vs. Ctrl, SMPDB analysis identified energy metabolism pathways among the most significantly enriched categories (Figure 2B), while KEGG enrichment emphasized metabolisms of glycerophospholipid, tryptophan, inositol phosphate, and nicotinate and nicotinamide, followed by glycolysis and gluconeogenesis (Supplementary Figure 1). This divergence in pathway enrichment suggests that while glucose metabolism remains central to AD pathology, additional metabolic pathways, particularly those linked to lipid signaling and neurotransmitter metabolism, may become increasingly disrupted.

The comparison of AD vs. MCI using SMPDB revealed that four of the top five enrichment pathways were energy-related (Figure 2C), whereas KEGG analysis identified two out of five pathways as energy metabolism-related (Supplementary Figure 1). Pathway impact analysis using SMPDB further supported energy metabolism as a central axis of disruption, with glycolysis, gluconeogenesis, Warburg effect, and pyruvate metabolism consistently emerging as the most significantly altered pathways (Figures 2D–F). Networks generated from the SMPDB enrichment analysis further emphasized energy pathways as central nodes of enrichment, underscoring their key role in metabolic alterations across disease states (Supplementary Figure 2). Collectively, these findings align with previous studies and further support the notion that dysregulated energy metabolism is a key driver of AD pathology and neurodegeneration (Patel et al., 2024; Butterfield and Halliwell, 2019).

### 3.3 Differential impact of sex on glucose metabolism and mitochondrial function in the visual cortex

Given the well-established sex-specific differences in AD prevalence, risk factors, and progression (Lopez-Lee et al., 2024), we investigated whether metabolic alterations differed between males and females across disease states. In this study, the Ctrl, MCI, and AD groups exhibited a comparable distribution of male and female subjects (Table 1).

**Figure 3:**
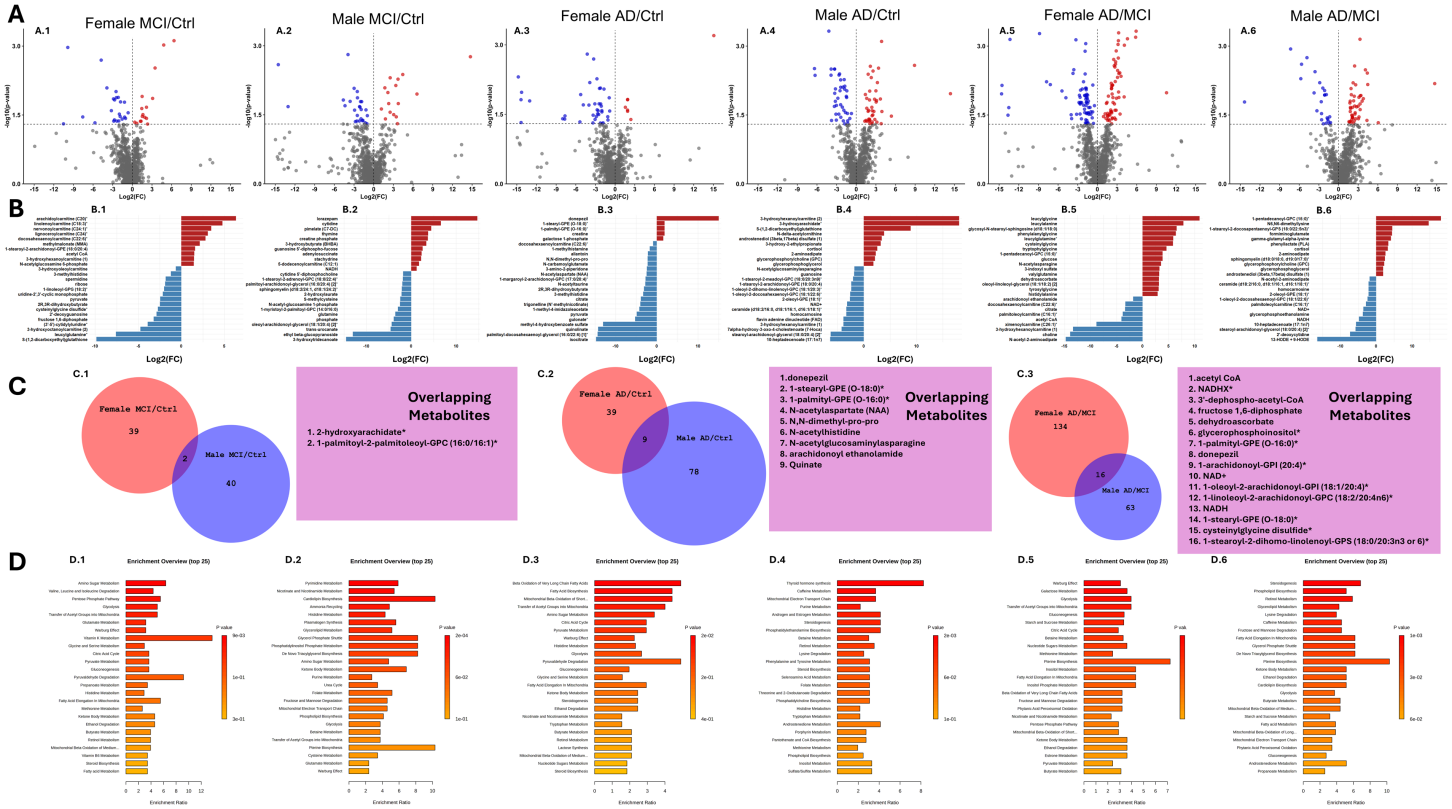
Differential impact of sex on glucose metabolism and mitochondrial function alterations in the visual cortex. Analyses were stratified by sex in comparisons of MCI/control (Ctrl), AD/Ctrl, and AD/MCI. **(A.1–A.6)** Volcano plots show differential metabolite levels, where the *Y*-axis represents −log10(*p*-value) from two-group *t*-tests, and the X-axis shows log2 fold change (Log2(FC)). Metabolites in blue are significantly decreased, while those in red are significantly increased *p* < 0.05. **(B.1–B.6)** The top 25 most significant metabolites (ranked by *p*-value) from the volcano plots above are displayed as Log2(FC). **(C.1–C.3)** Venn diagrams depict the number of altered metabolites between sexes and their overlap. **(D.1–D.6)** The altered metabolites metabolite set enrichment analysis using the Small Molecule Pathway Database (SMPDB) identifies key metabolic pathways affected in each comparison. Statistical significance of metabolite ratios was determined using two-group *t*-tests *p* < 0.05.

To identify sex-specific changes, two-group t-test comparisons were performed separately for males and females. Volcano plots highlight significantly altered metabolites for MCI vs. Ctrl (Figures 3A.1,A.2), AD vs. Ctrl (Figures 3A.3,A.4), and AD vs. MCI (Figures 3A.5,A.6) within each sex. In addition, the top 25 most significantly altered metabolites for each comparison are ranked by log 2-fold-change and displayed as bar plots (Figure 3B). Across comparisons, we observed consistent involvement of metabolites related to central energy metabolism, including glucose, pyruvate, NADH, and acetyl-CoA, suggesting altered mitochondrial function and glycolytic metabolism. In parallel, several acylcarnitines (e.g., palmitoylcarnitine, arachidonoylcarnitine) and phosphatidylcholine species (e.g., 1-palmitoyl-GPC, 1-stearoyl-GPC) were also altered, implicating modified lipid metabolism and potential shifts in substrate utilization. Additional changes in glutathione disulfide and gamma-glutamyl peptides indicate alterations in redox balance and oxidative stress regulation (Zhang et al., 2012).

To examine the overlap in metabolic changes, Venn diagrams were generated (Figure 3C). In MCI vs. Ctrl and AD vs. Ctrl comparisons, overlap was limited (two and nine shared metabolites, respectively) between female and male subjects. In contrast, the AD vs. MCI comparison revealed greater convergence of 16 metabolites, including glucose, NADH, acetyl-CoA, and glutathione-related metabolites. Pathway enrichment analysis (SMPDB) further underscored sex-specific metabolic dysregulation (Figure 3D). Enrichment profiles differed by sex: females displayed more substantial enrichment in energy-related pathways, across MCI and AD disease states, these pathways include Glycolysis, Gluconeogenesis, Transfer of Acetyl Groups into Mitochondria, and the Warburg Effect. Whereas males exhibited more diffuse enrichment profiles in the MCI disease state encompassing multiple lipid pathways such as Cardiolipin biosynthesis, Plasmalogen Synthesis, Glycerolipid Metabolism, and *De Novo* Triacylglycerol Metabolism (Figure 3D). Male AD subjects had higher enrichment of hormone-related pathways compared to other groups, but their enrichment profiles still included key mitochondria related pathways such as Nicotinate and Nicotinamide Metabolism, Fatty Acid Elongation in Mitochondria, Mitochondrial Electron Chain, and Mitochondrial Beta-Oxidation of Short Chain Fatty Acids. Repeated pathway enrichment analysis using the KEGG database revealed similar enrichment pathways diverging in the MCI group by sex and reconverging during AD (Supplementary Figure 3).

### 3.4 Aberrant glucose metabolism in the visual cortex of AD and MCI subjects

**Figure 4:**
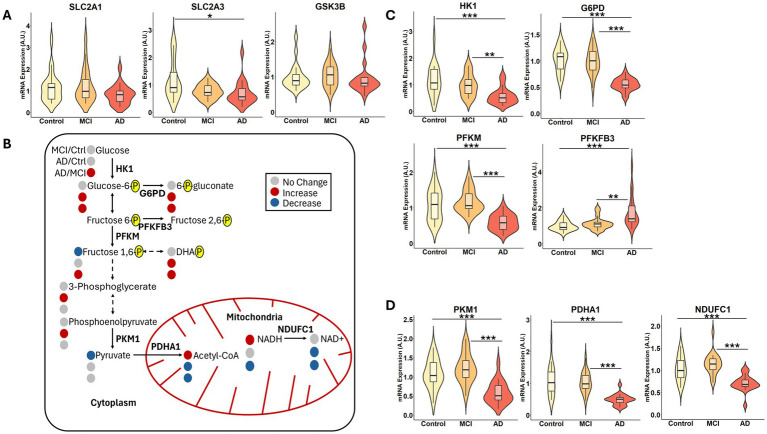
Aberrant glucose metabolism is present in the visual cortex of AD and MCI subjects. **(A)** mRNA expression levels of *SLC2A1*, *SLC2A3*, and *GSK3B* in control (Ctrl), MCI, and AD samples. **(B)** Schematic representation of selected metabolites from untargeted metabolomic analysis involved in glycolysis and related pathways, displaying metabolite ratios for MCI/Ctrl, AD/Ctrl, and AD/MCI, where red indicates significantly increased, blue indicates significantly decreased, and grey indicates no statistically significant difference. Enzymes with measured mRNA expression are indicated in the schematic. mRNA expression levels of **(C)** *HK1*, *G6PD*, *PFKM*, *PFKFB3*, and **(D)** *PKM1*, *PDHA1*, *NDUFC1* across disease states. Statistical significance of metabolite ratios was determined using two-group *t*-tests. Statistical significance of gene expression was determined using the Kruskal-Wallis test followed by Dunn’s *post hoc* test with Benjamini-Hochberg multiple comparisons adjustment. Gene expression analyses were performed on *n* = 19–20 per group, and the relative fold changes were expressed in arbitrary units (A.U.). Significance is indicated as *p* < 0.05 (*), *p* < 0.01 (**), and *p* < 0.001 (***).

Given that glycolysis and gluconeogenesis were consistently among the most altered pathways in both MCI and AD subjects compared to controls, we further investigated changes in glucose metabolism and potential mechanisms underlying these disruptions. We began by assessing the gene expression of transporters that regulate cellular glucose availability. While *SLC2A1* (GLUT1) expression was not significantly altered across groups, *SLC2A3* (GLUT3) was significantly reduced in the AD group, suggesting impaired neuronal glucose uptake (Figure 4A). Additionally, *SLC2A4* (GLUT4) expression was undetectable (data not shown), consistent with its minimal and region-specific expression in the brain. Also, we examined glycogen synthase kinase 3 beta (*GSK3B*), a regulator of glycogen metabolism and insulin signaling implicated in AD pathogenesis and a therapeutic target (Zhao et al., 2024). However, expression levels did not significantly differ across groups (Figure 4A), suggesting that, in the visual cortex, *GSK3B* transcriptional changes may not contribute to the observed metabolic dysfunction.

A schematic overview (Figure 4B) illustrates glycolysis and mitochondrial-related metabolites detected in our studies, along with their relative alterations across groups. The AD group consistently exhibited higher concentrations of glycolytic intermediates (downstream of glucose until reaching pyruvate) compared to both the control and MCI groups. In contrast, acetyl-CoA, NADH, and NAD + were decreased in AD relative to both groups. The MCI group showed increased levels of acetyl-CoA and NADH compared to controls, potentially indicating a compensatory metabolic response.

To determine if altered glucose metabolism could be partially explained by altered gene transcription, we analyzed the expression of rate-limiting enzymes in glycolysis. Several glycolytic regulators, including hexokinase 1 (*HK1*) and phosphofructokinase (*PFKM*), were significantly downregulated in AD, alongside glucose 6-phosphate dehydrogenase (*G6PD*), a key regulatory enzyme of the pentose phosphate pathway, implicating disrupted glucose flux and redox homeostasis through G6PD’s generation of metabolites essential for regeneration of NADPH (Figure 4C). Notably, 6-phosphofructo-2-kinase/fructose-2,6-biphosphatase (*PFKFB3*) was increased in AD, potentially reflecting a compensatory response aimed at maintaining glycolytic activity. Further downstream, we observed lower expression levels of pyruvate kinase (*PKM1*) in AD subjects, along with reduced expression of *PDHA1*, a subunit of pyruvate dehydrogenase, and *NDUFC1*, a subunit of NADH dehydrogenase, the first complex in the electron transport chain (Figure 4D).

Next, the gene expression of glucose transporters and downstream enzymes was stratified by sex, which revealed that AD females had a higher expression of *PFKFB3* than AD males (Supplementary Figure 4). Females in the control group also had higher expression of *PFKM*. This highlights that disrupted glucose metabolism appears to be universally present across both sexes, but no other gene expression was significantly altered in disease states. Together, these findings indicate a broad dysregulation of glucose metabolism and mitochondrial function in the visual cortex of AD subjects, consistent with reports of metabolic dysfunction in AD.

### 3.5 Expression of key modulators of energy balance is altered in the visual cortex of AD subjects

**Figure 5:**
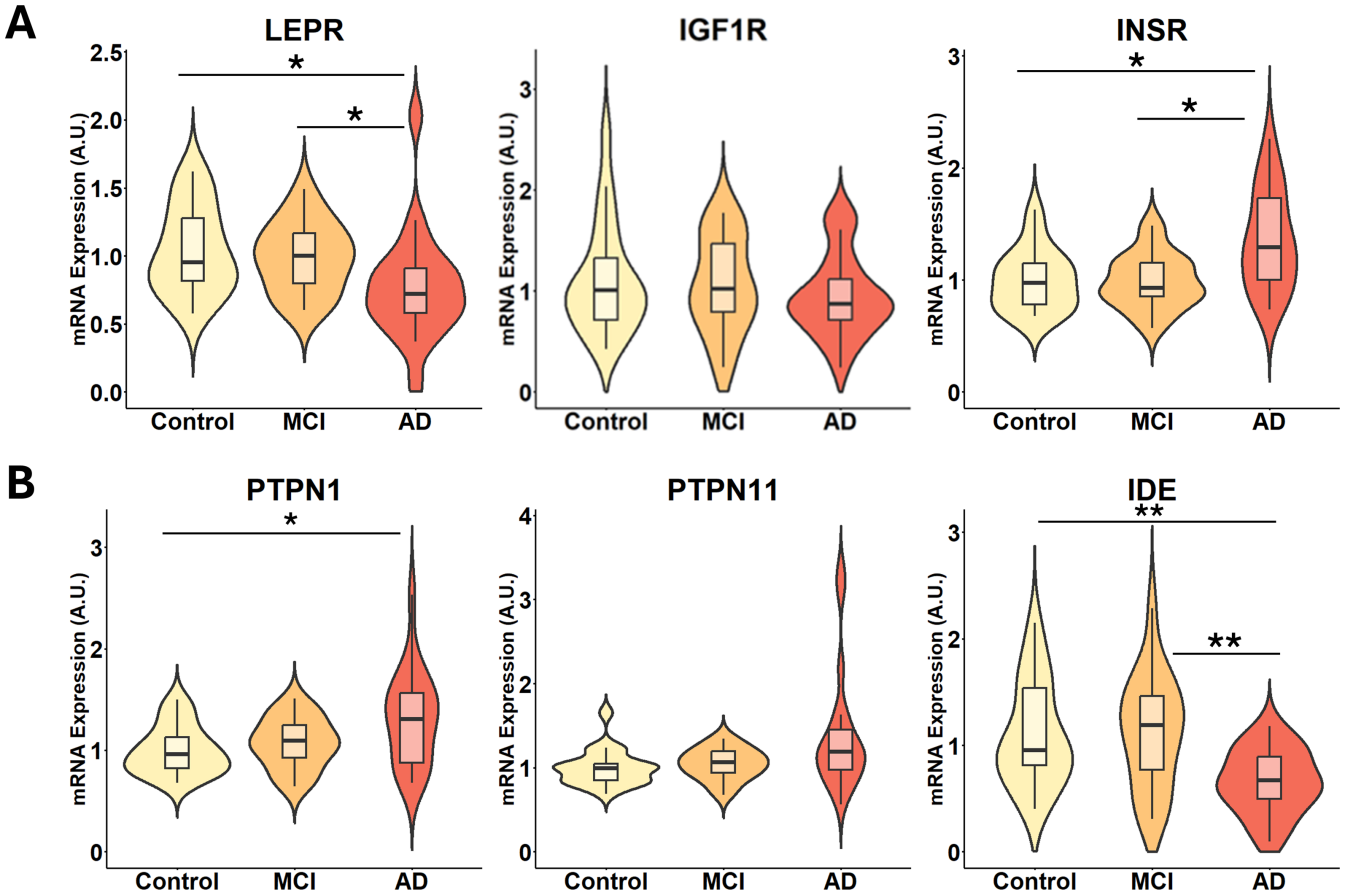
Expression of key regulators of energy regulation is altered in the visual cortex of AD subjects. mRNA expression levels of **(A)** *LEPR, IGF1R, INSR*, and **(B)** *PTPN1, PTPN11, IDE* in the visual cortex samples from control, MCI and AD subjects. Statistical significance was determined using the Kruskal–Wallis test followed by Dunn’s *post hoc* test with Benjamini-Hochberg multiple comparisons adjustment. *n* = 18–20 per group, and the relative fold changes were expressed in arbitrary units (A.U.). Significance is indicated as *p* < 0.05 (*) and *p* < 0.01 (**).

To further characterize disruptions in energy metabolism, we examined the expression of key modulators of energy metabolism through insulin and leptin signaling pathways. Notably, the expression of the leptin receptor (*LEPR*) was significantly reduced in AD subjects, whereas the insulin-like growth factor 1 receptor (*IGF1R*) levels were not significantly changed (Figure 5A). While counterintuitive, increased insulin receptor (IR encoded by *INSR*) expression may reflect a compensatory response to impaired downstream insulin signaling (Figure 5A). Stratification by sex did not reveal disease-specific differences in *INSR* expression; however, male controls exhibited higher baseline *INSR* levels than females (Supplementary Figure 5).

To assess downstream leptin and insulin signaling, we evaluated the expression of protein tyrosine phosphatases that are established negative-regulators of these pathways, namely protein tyrosine phosphatase 1B (PTP1B, encoded by *PTPN1*) and the Src homology phosphatase 2 (Shp2, encoded by *PTPN11*) (Banno et al., 2010; Zhao et al., 2022). *PTPN1* was significantly upregulated in AD, suggesting potential alterations in IR phosphorylation, while *PTPN11* expression was not significantly changed (Figure 5B). Additionally, we examined the insulin-degrading enzyme (*IDE*), a protease that regulates both insulin and Aβ clearance and has been proposed as a mechanistic link between diabetes and AD (Farris et al., 2003). Consistent with this hypothesis, *IDE* was significantly downregulated in AD, reinforcing a potential feedback loop between impaired insulin clearance and Aβ accumulation (Figure 5B). These findings demonstrate that, in addition to impaired metabolic processing (Figure 4), several proteins involved in regulating energy balance are also dysregulated in the AD visual cortex, indicating a reduced capacity to coordinate metabolic function.

### 3.6 Gene expression of markers of inflammation and Alzheimer’s disease is increased in AD subjects

**Figure 6:**
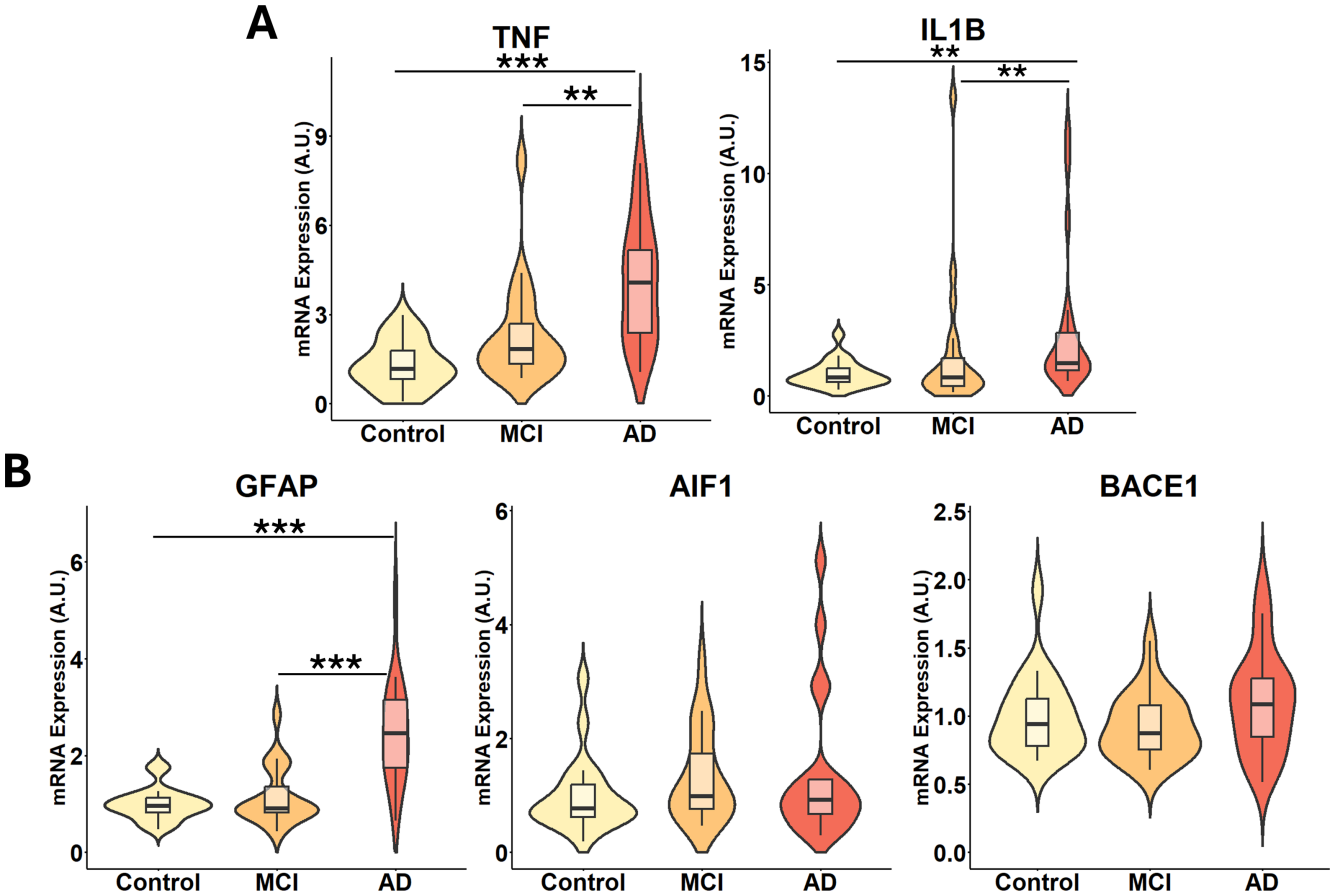
Gene expression of markers of inflammation and Alzheimer’s disease is increased in AD subjects. Gene expression levels of **(A)** inflammatory signaling molecules *TNF, IL1B*, and **(B)** Alzheimer’s disease markers *GFAP, AIF1, BACE1* in the visual cortex samples from control, MCI and AD subjects. Statistical significance was determined using the Kruskal-Wallis test followed by Dunn’s *post hoc* test with Benjamini-Hochberg multiple comparisons adjustment. *N* = 20 per group and the relative fold changes were expressed in arbitrary units (A.U.). Significance is indicated as *p* < 0.01 (**) and *p* < 0.001 (***).

To assess neuroinflammation and AD-related pathology, we quantified the expression of key inflammatory markers and AD-associated genes in the visual cortex across disease stages. Pro-inflammatory cytokines, tumor necrosis factor alpha (*TNF*) and interleukin-1 beta (*IL1B*) were significantly upregulated in AD compared to both control and MCI groups (Figure 6A), indicating an enhanced inflammatory response with disease progression. Similarly, the expression of the glial fibrillary acidic protein (*GFAP*), a marker of astrocyte reactivity, was elevated in both MCI and AD, with a further increase in AD, consistent with progressive astrocytic activation (Kamphuis et al., 2014). In contrast, the expression of allograft inflammatory factor 1 (*AIF1*), a marker of microglial activation, was not significantly altered (Figure 6B).

To assess alterations in pathways linked to amyloid pathology, we measured beta-secretase 1 (*BACE1*), as this enzyme is responsible for amyloid precursor protein cleavage. *BACE1* expression was unaltered across groups (Figure 6B), consistent with limited transcriptional regulation of amyloidogenic processing in the visual cortex at this stage of the disease. None of the inflammatory genes showed differential expression when stratified by sex (Supplementary Figure 6). These findings highlight a strong inflammatory response at the gene expression level in the AD visual cortex, likely driven by astrocytic activation and increased cytokine expression.

### 3.7 Gene expression in the visual cortex correlates with Braak staging

**Figure 7:**
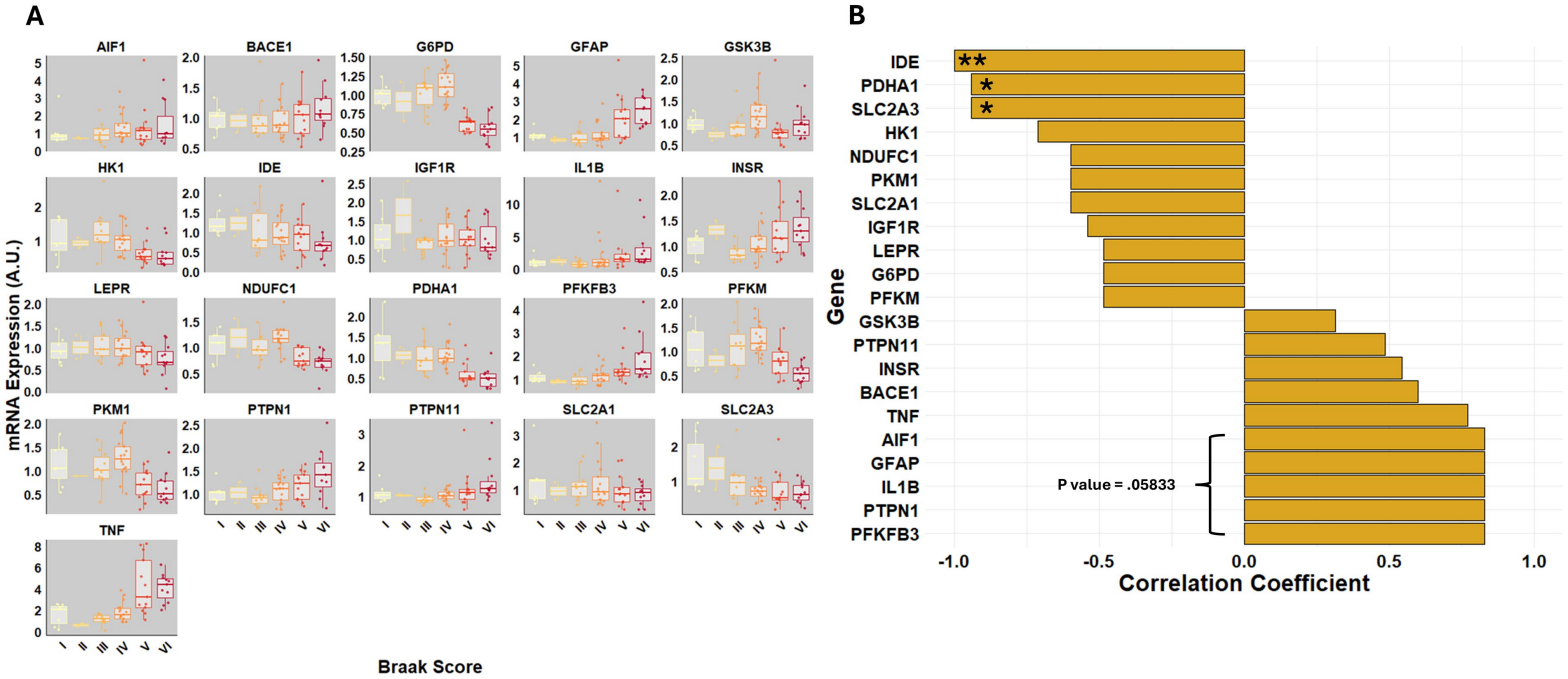
Gene expression correlates with Braak staging in the visual cortex. **(A)** Box plots show mRNA expression levels of metabolic and inflammatory genes across Braak stages I–VI. Relative mRNA fold changes were expressed in arbitrary units (A.U.). **(B)** Spearman’s correlation coefficients between gene expression and Braak stages are shown as a ranked bar plot, where positive correlations indicate increased expression with disease progression, and negative correlations indicate decreased expression.

Braak staging is a neuropathological framework used to classify the progression of AD based on the distribution and severity of tau pathology. Higher Braak stages (IV–VI) indicate widespread neurofibrillary tangle accumulation and are strongly associated with cognitive decline (Braak and Braak, 1991; Biel et al., 2021). Given its relevance in defining disease severity, we investigated whether gene expression changes in the visual cortex correlate with the Braak stage (Figure 7A).

To systematically assess these relationships, a Spearman correlation was performed between Braak stage and the expression of key genes involved in glucose metabolism, mitochondrial function, insulin signaling, and neuroinflammation. Several metabolic genes exhibited a strong negative correlation with disease progression, including *IDE*, *PDHA1*, and the neuronal glucose transporter *SLC2A3* (Figure 7B), suggesting a progressive reduction in energy metabolism and regulation with increasing tau pathology. Additionally, *HK1*, *PKM1*, and *NDUFC1*, which regulate glycolysis and mitochondrial function, showed a downward trend in expression with increasing Braak stage but did not reach significance. (Figure 7B).

In contrast, inflammatory markers such as *TNF*, *IL1B*, and *GFAP* showed a trend toward increasing expression with advancing pathology (Figure 7B). However, these correlations did not reach statistical significance. Both *PTPN1* and *PFKFB3* trended upward without reaching significance as well. Few metabolites correlated with Braak stage; only donepezil, a common AD treatment, had a Spearman coefficient > 0.5 (Supplementary Figure 7). Together, these findings reinforce the progressive impairment of metabolic homeostasis as tau pathology increases, accompanied by elevated inflammation in the visual cortex, with a marked decline in genes essential for neuronal energy metabolism.

## 4 Discussion

### 4.1 Metabolic reprogramming in the visual cortex of AD and MCI subjects

Metabolic dysfunction is a central feature of AD (Demetrius et al., 2021), yet its manifestation in the visual cortex remains underexplored. Using untargeted metabolomics, we identified widespread metabolic alterations in AD and MCI subjects, with 26 metabolites significantly altered in MCI/Ctrl, 108 in AD/Ctrl, and 173 in AD/MCI. The greater number of metabolites altered between AD and MCI compared with AD and controls likely indicate progressive metabolic divergence along the disease spectrum. Compensatory hypermetabolism can occur in the early stage of MCI (Bakhtiari et al., 2024; Merlo et al., 2019), but this phenomenon is not examined in depth here. However, elevations in NADH and acetyl-CoA in the MCI group suggest early mitochondrial engagement, while increased glycolytic intermediates in AD may reflect a decoupling of glycolysis from oxidative metabolism as the disease progresses (Figure 4). Hierarchical clustering of the top 25 metabolites showed limited separation between MCI and Ctrl samples, whereas AD subjects clustered distinctly, suggesting a more pronounced metabolic phenotype. The more subtle metabolic shifts in MCI compared with AD are in keeping with early-stage metabolic dysregulation that precedes the more robust alterations observed in dementia.

Our findings support that energy metabolism is among the first pathways disrupted in MCI, offering a potential therapeutic window. Dysregulation of glucose metabolism was prominent in both MCI and AD. Indeed, pathway enrichment analyses identified Glycolysis, Pyruvate Metabolism, Gluconeogenesis, and the Warburg Effect as the most significantly altered pathways in AD and MCI subjects. Notably, these same pathways emerged across both SMPDB and KEGG analyses, reinforcing the rigor of the analyses. Because central metabolic pathways are tightly regulated, individual metabolites may not rank among the most discriminatory features, whereas pathway-level enrichment captures coordinated metabolic disruptions.

Enrichment networks were generated, and within these networks, the most prominent and highly interconnected nodes represented core metabolic pathways, highlighting their central role in the broader pattern of biochemical disruption (Supplementary Figure 2). These findings in the visual cortex align with prior reports of disrupted glucose and energy metabolism across other brain regions in AD (Mosconi, 2005; Gonzalez et al., 2022).

To explore the molecular underpinnings underlying metabolic alterations, we assessed the gene expression of key targets regulating glucose availability and metabolism. At the transporter level, *SLC2A3* (GLUT3) was significantly reduced in AD subjects, implicating impaired neuronal glucose uptake (Kyrtata et al., 2021). *SLC2A1* (GLUT1) remained unchanged across disease states, and *SLC2A4* (GLUT4) was undetectable, consistent with its limited role in areas outside the hypothalamus and other energy-regulating centers of the brain (Szablewski, 2017). Downregulation of *HK1*, *PFKM*, and *PKM1*, three rate-limiting enzymes in glycolysis, suggests a reduced capacity for glycolytic flux in AD. Expression of *G6PD*, the regulator of the pentose phosphate pathway, was reduced in the AD group. Overexpressing *G6PD* in an AD mouse model improved metabolism and reduced oxidative stress, yielding cognitive protection (Correas et al., 2024).

AD subjects showed mitochondrial dysfunction, with reduced *PDHA1* and acetyl-CoA, indicating impaired pyruvate entry into the TCA cycle. Concurrently, *NDUFC1* expression as well as NAD+, nicotinamide ribonucleotide, flavin adenine dinucleotide, the TCA cycle intermediates citrate and isocitrate levels were all reduced in the AD subjects compared to Controls, suggesting deficits in electron transport chain activity and diminished oxidative phosphorylation capacity (Supplementary data sheet 2). These findings are consistent with evidence linking mitochondrial dysfunction to AD pathogenesis (Wang et al., 2020; Fang et al., 2019). Moreover, *PFKFB3* expression was upregulated in the AD group. Upregulation of PFKFB3, an important regulator of glycolysis, has been shown to be induced by both shifting energy demands and Aβ (Li et al., 2019; Wang et al., 2023; Woo et al., 2025). This upregulation has been associated with a glycolysis-biased metabolic reprogramming in neurodegenerative disease models through PFKFB3’s ability to generate fructose-2,6-bisphosphate, a potent activator of PFKM (Rodriguez-Rodriguez et al., 2012). Downregulation of *HK1*, *PFKM*, and *PDHA1* in AD visual cortex mirrors oligodendrocyte-specific impairments reported in RNA-seq meta-analyses of AD brains (Saito et al., 2021). Though not cell-type specific, the overlap points toward glial contributions to AD-linked hypometabolism. Overall, our findings reinforce the concept that metabolic shifts intensify with disease progression, as observed in other brain regions (Butterfield and Halliwell, 2019). Together, the elevated levels of upstream glycolytic intermediates in AD are consistent with impaired downstream mitochondrial utilization of glycolytic carbon. This interpretation is supported by reduced expression of *PDHA1* and *NDUFC1*, decreased acetyl-CoA TCA cycle intermediates, and NAD(H) levels, and negative correlations between key metabolic genes and Braak stage. Collectively, these findings suggest a bottleneck at mitochondrial entry rather than activation of glycolysis.

### 4.2 Sex-specific metabolic alterations in AD

Sex differences in AD pathology are well-documented, with women exhibiting higher prevalence rates and faster cognitive decline than men (Demetrius et al., 2021; Mielke, 2018). In this study, sex-stratified analyses revealed divergent metabolic profiles across disease stages. Female MCI subjects showed greater enrichment in glycolysis, the transfer of acetyl groups into mitochondria, and the pentose phosphate pathway, in line with early energy and redox disruptions. In contrast, males with MCI exhibited greater enrichment in lipid-related pathways, including Glycerolipid Metabolism, Phospholipid Biosynthesis, and Ketone Body Metabolism, consistent with altered membrane composition and energy storage. By the AD stage, both sexes exhibited widespread metabolic disruptions, although females continued to display more focused changes in glucose metabolism and antioxidant pathways. Males demonstrated broader involvement of mitochondrial and steroid hormone–related pathways. Despite these differences, mitochondrial dysfunction emerged as a common feature across sexes, with shared reductions in NADH, acetyl-CoA, and NAD + observed in the AD vs. MCI comparison, indicating convergence of central energy failure. These findings suggest sex shapes AD metabolic progression, with early glucose and lipid responses differing but converging on shared mitochondrial deficits. While these patterns provide insight into sex-related differences in metabolic flexibility or vulnerability, the reduced sample size in sex-stratified analyses may limit the resolution of more subtle or nuanced changes. Future studies with larger cohorts are necessary to expand on these observations, particularly in the context of personalized metabolic interventions for AD.

While few genes were sex-differentially expressed in our dataset, Park et al. (2023) reported sex-specific alterations in glucose metabolism–related genes in AD. In our dataset, key glycolytic genes were downregulated in AD, but not sex-dependent. However, *PFKFB3* exhibited significant sex-specific upregulation in the female AD group (Supplementary Figure 4). Of note, Park et al. similarly reported female-specific upregulation of *PFKFB3*, underscoring this gene as a consistent marker of sex-dependent metabolic reprogramming. These findings highlight the importance of accounting for sex-specific metabolic pathways in early detection and treatment, as distinct glucose and lipid patterns may shape AD progression and therapy response.

### 4.3 Dysregulated insulin signaling and the potential role of insulin resistance in AD

Insulin resistance is recognized as a contributing factor in AD pathogenesis (Kshirsagar et al., 2021). We observed elevated *INSR* expressions in AD, contrasting prior reports, possibly due to regional brain differences (Steen et al., 2005; Zhu et al., 2005). Conversely, *LEPR* expression was significantly reduced, potentially disrupting energy balance via leptin’s roles in appetite, plasticity, and neuroprotection (Harvey, 2007).

Further supporting the presence of insulin resistance in AD is the upregulation of the prototypical protein tyrosine phosphatase PTP1B. PTP1B regulates multiple energy-sensing pathways through its ability to dephosphorylate key targets in the insulin and leptin signaling pathways. Of note, whole-body and neuron-specific genetic deletion of PTP1B leads to a leaner phenotype in mice fed a high fat diet (Zhao et al., 2022; Elchebly et al., 1999; Klaman et al., 2000). PTP1B’s role in neuronal energy homeostasis makes it a potential therapeutic target in neurodegeneration (Zhao et al., 2022; Bence et al., 2006). Indeed, neuronal disruption or pharmacological inhibition of PTP1B yields beneficial effects in pre-clinical mouse models of AD (Ricke et al., 2020; Franklin et al., 2025; Cen et al., 2025; Vieira et al., 2017). Additionally, the expression of *IDE* was significantly reduced in AD brains. *IDE* plays a dual role in degrading both insulin and Aβ. A mouse model of AD with a genetic deletion of *IDE* showed both increased cerebral Aβ accumulation and hyperinsulinemia (Farris et al., 2003). Therefore, *IDE*’s downregulation may exacerbate both insulin resistance and Aβ accumulation, further reinforcing the link between metabolic dysfunction and AD pathology.

### 4.4 Neuroinflammation and AD pathology in the visual cortex

Chronic neuroinflammation is a hallmark of AD (Heneka et al., 2025). In our study, *TNF* and *IL1B* were significantly upregulated in AD subjects, highlighting the presence of a pro-inflammatory milieu in the visual cortex. Cortisol was a top discriminatory metabolite, separating AD subjects from MCI and Ctrl subjects in the hierarchical clustering analysis (Figures 1E,F). This aligns with findings that higher cerebrospinal fluid cortisol correlates with global decline and elevated tau in AD and MCI (Ouanes et al., 2022). *GFAP* expression was increased in MCI and AD, consistent with reactive astrocytosis in AD (Kamphuis et al., 2014). *AIF1* expression was not significantly increased in the AD visual cortex. However, AIF1 is usually assessed at the protein level via immunohistochemistry, mRNA transcript levels may not fully reflect microglial activation status (Hopperton et al., 2018).

### 4.5 Progressive metabolic dysfunction correlates with Braak staging

Braak staging is a neuropathological classification that reflects the progression of tau pathology and is strongly correlated with cognitive decline (Braak and Braak, 1991; Biel et al., 2021). We found strong negative correlations between Braak stage and expression of metabolic genes (*IDE*, *PDHA1*, *SLC2A3*), reinforcing that glucose metabolism declines with advancing tau pathology. Pro-inflammatory markers (*TNF*, *IL1B*, *GFAP*) trended higher with Braak stage but were not statistically significant. This suggests metabolic dysfunction closely tracks tau burden, while neuroinflammation may be regionally variable or delayed in the visual cortex.

### 4.6 Study limitations

This study has several limitations. The use of post-mortem human tissue provides a cross-sectional snapshot and precludes direct assessment of metabolic flux or causality. In addition, gene expression changes may not directly reflect protein abundance or enzymatic activity, and future studies incorporating proteomic or functional assays will be important to further resolve these relationships. Metabolomic and transcriptional analyses were performed on bulk cortical tissue and thus cannot fully account for cell-type–specific contributions. Finally, the cohort consisted exclusively of donors of Caucasian ancestry, which may limit generalizability to more diverse populations.

## 5 Conclusion

Our findings strengthen the growing consensus that impaired energy metabolism plays a central role in AD. Importantly, the observation of substantial metabolic dysregulation in the relatively preserved visual cortex suggests that these alterations may arise early in disease progression, prior to extensive neurodegeneration. This supports the rationale for targeting metabolic dysfunction during prodromal or early disease stages, when interventions may be most effective. These findings highlight the therapeutic potential of metabolic interventions such as dietary modification, physical activity, or therapeutics that enhance insulin sensitivity as strategies to delay or modify disease progression (Kazibwe et al., 2024; Ahlskog et al., 2011; Barnard et al., 2014; Zotcheva et al., 2018). Promising evidence is emerging from multiple approaches. Long-term intranasal insulin has been shown to improve memory outcomes in AD patients (Hallschmid, 2021) however additional studies are warranted (Craft et al., 2020; Smith et al., 2025), and glucagon-like peptide receptor agonists are advancing in clinical trials (Edison et al., 2026; Tang et al., 2025; Wang et al., 2025; Nørgaard et al., 2022). Taken together, these insights position metabolic resilience as a promising frontier in AD therapy, and it is likely that improving metabolic brain function will be an increasingly important component of the AD therapeutic landscape.

## Data Availability

The raw data supporting the conclusions of this article will be made available by the authors, without undue reservation.
