# Supplementary material for: Dysregulated glucose metabolism in the visual cortex of human subjects with mild cognitive impairment and Alzheimer’s disease
Source: Front Aging Neurosci. 2026 Apr 9;18:1710075. doi: 10.3389/fnagi.2026.1710075 (PMC13102864; doi:10.3389/fnagi.2026.1710075)
Supplement: Supplementary file 1 [file Table_1.docx]

| **Appendix Table 1. Demographic Data and Neuropathologic Diagnosis of Subjects** | | | | | | |  |  |  |
| --- | --- | --- | --- | --- | --- | --- | --- | --- | --- |
| **ID** | **Group** | **Sex** | **Age** | **Braak Score** | **PMI** | **APOE** |  |  |  |
| 07-37 | Ctrl | Male | 89 | II | 5.5 | 3,3 |  |  | **Average (PMI)** |
| 07-73 | Ctrl | Female | 76 | I | 4 | 3,4 |  | **Ctrl** | 3.443 |
| 08-55 | Ctrl | Male | 71 | I | 3 | 3,3 |  | **MCI** | 3.651 |
| 08-77 | Ctrl | Female | ≥90 | IV | 7.25 | 3,3 |  | **AD** | 3.4235 |
| 09-20 | Ctrl | Female | ≥90 | III | 3.5 | 3,3 |  |  |  |
| 09-45 | Ctrl | Female | 84 | III | 3 | 3,3 |  |  |  |
| 09-50 | Ctrl | Male | 53 | I | 3.66 | 3,3 |  |  |  |
| 09-57 | Ctrl | Male | 80 | III | 3.5 | 2,3 |  |  |  |
| 10-07 | Ctrl | Male | 89 | IV | 3.5 | 3,3 |  |  |  |
| 10-26 | Ctrl | Female | ≥90 | III | 2.5 | 3,3 |  |  |  |
| 10-39 | Ctrl | Male | ≥90 | I | 3 | 3,3 |  |  |  |
| 10-63 | Ctrl | Male | 79 | II | 3 | 3,3 |  |  |  |
| 10-70 | Ctrl | Male | 74 | I | 3.25 | 2,3 |  |  |  |
| 11-28 | Ctrl | Female | 79 | III | 2.22 | 2,3 |  |  |  |
| 15-46 | Ctrl | Female | 82 | I | 2.07 | 2,3 |  |  |  |
| 16-40 | Ctrl | Female | ≥90 | III | 2.67 | 2,3 |  |  |  |
| 16-51 | Ctrl | Male | ≥90 | III | 2.83 | 3,4 |  |  |  |
| 17-50 | Ctrl | Male | ≥90 | I | 3.41 | 3,3 |  |  |  |
| 18-16 | Ctrl | Female | 84 | III | 3 | 3,3 |  |  |  |
| 18-42 | Ctrl | Female | 87 | III | 4 | 3,4 |  |  |  |
| 07-52 | MCI | Female | ≥90 | IV | 2.45 | 3,3 |  |  |  |
| 08-52 | MCI | Male | ≥90 | IV | 2.5 | 3,3 |  |  |  |
| 10-78 | MCI | Female | ≥90 | IV | 3.16 | 2,3 |  |  |  |
| 11-07 | MCI | Female | ≥90 | IV | 3.16 | 3,3 |  |  |  |
| 11-96 | MCI | Male | 82 | VI | 5 | 3,4 |  |  |  |
| 13-61 | MCI | Male | 86 | IV | 3.16 | 3,3 |  |  |  |
| 14-36 | MCI | Female | 82 | IV | 3.17 | 2,3 |  |  |  |
| 14-47 | MCI | Female | 77 | IV | 2.63 | 3,4 |  |  |  |
| 15-16 | MCI | Male | ≥90 | IV | 4 | 3,3 |  |  |  |
| 15-18 | MCI | Female | ≥90 | IV | 2.57 | 3,3 |  |  |  |
| 15-35 | MCI | Female | 80 | IV | 5.77 | 2,3 |  |  |  |
| 15-73 | MCI | Male | 84 | V | 2.2 | 3,4 |  |  |  |
| 15-80 | MCI | Female | ≥90 | IV | 3.68 | 2,3 |  |  |  |
| 17-15 | MCI | Female | ≥90 | IV | 3.15 | 3,3 |  |  |  |
| 17-43 | MCI | Female | ≥90 | IV | 2.52 | 3,3 |  |  |  |
| 20-11 | MCI | Female | ≥90 | IV | 7.65 | 3,3 |  |  |  |
| 20-61 | MCI | Female | ≥90 | IV | 2.83 | 3,3 |  |  |  |
| 20-64 | MCI | Female | ≥90 | V | 2.92 | 3,4 |  |  |  |
| 20-65 | MCI | Male | ≥90 | III | 3.07 | 3,3 |  |  |  |
| 20-69 | MCI | Male | ≥90 | V | 7.43 | 3,4 |  |  |  |
| 06-09 | AD | Female | ≥90 | V | 3.5 | 3,3 |  |  |  |
| 10-36 | AD | Male | ≥90 | V | 4.5 | 3,3 |  |  |  |
| 11-27 | AD | Male | 89 | V | 2.2 | 2,3 |  |  |  |
| 11-35 | AD | Female | ≥90 | V | 2.83 | 3,3 |  |  |  |
| 11-59 | AD | Male | 83 | V | 3 | 4,4 |  |  |  |
| 12-27 | AD | Female | 89 | VI | 2.7 | 3,3 |  |  |  |
| 13-10 | AD | Male | 74 | VI | 2.58 | 3,4 |  |  |  |
| 13-31 | AD | Male | 85 | VI | 3.58 | 3,4 |  |  |  |
| 13-42 | AD | Male | 80 | VI | 3 | 4,4 |  |  |  |
| 13-53 | AD | Female | ≥90 | VI | 3.73 | 3,4 |  |  |  |
| 13-54 | AD | Female | 85 | VI | 3.42 | 3,3 |  |  |  |
| 13-66 | AD | Male | 75 | V | 3.83 | 3,4 |  |  |  |
| 13-75 | AD | Male | 77 | VI | 3.62 | 3,4 |  |  |  |
| 14-06 | AD | Male | 85 | V | 3.5 | 3,4 |  |  |  |
| 14-11 | AD | Male | 82 | V | 3.98 | 2,4 |  |  |  |
| 14-48 | AD | Male | 73 | VI | 4.83 | 3,4 |  |  |  |
| 15-03 | AD | Female | 81 | VI | 3.85 | 3,4 |  |  |  |
| 15-19 | AD | Female | 83 | V | 3.62 | 3,3 |  |  |  |
| 15-27 | AD | Female | 84 | VI | 3.33 | 3,3 |  |  |  |
| 20-66 | AD | Female | 85 | V | 2.87 | 3,4 |  |  |  |
