# Supplementary material for: Dysregulated glucose metabolism in the visual cortex of human subjects with mild cognitive impairment and Alzheimer’s disease
Source: Front Aging Neurosci. 2026 Apr 9;18:1710075. doi: 10.3389/fnagi.2026.1710075 (PMC13102864; doi:10.3389/fnagi.2026.1710075)
Supplement: Supplementary file 2 [file Table_2.docx]

| Primer Table | | | | | |  |  |  |  |  |
| --- | --- | --- | --- | --- | --- | --- | --- | --- | --- | --- |
| Gene |  | Sequence (5'-3') | Product (bp) | Accession number | Reference |  |  |  | Refrence |  |
| UBE2D2 | F | TGCCTGAGATTGCTCGGATCT | 81 | NM_003339.3 | 1 |  |  |  | 1 | <https://doi.org/10.1038/srep37116> |
|  | R | TCGCATACTTCTGAGTCCATTCC |  |  |  |  |  |  | 2 | <https://doi.org/10.1038/cddis.2014.292> |
| SLC2A3 | F | GCTGGGCATCGTTGTTGGA | 123 | NM_006931.3 |  |  |  |  | 3 | https://doi.org/10.1371/journal.pone.0057610 |
|  | R | GCACTTTGTAGGATAGCAGGAAG |  |  |  |  |  |  | 4 | <https://doi.org/10.1186/s12929-017-0383-3> |
| PFKM | F | GGTGCCCGTGTCTTCTTTGT | 99 | NM_001166686.2 |  |  |  |  | 5 | https://doi.org/10.3389/fonc.2021.709044 |
|  | R | AAGCATCATCGAAACGCTCTC |  |  |  |  |  |  |  |  |
| PFKFB3 | F | CAGTTGTGGCCTCCAATATC | 113 | NM_001282630.3 | 2 |  |  |  |  |  |
|  | R | GGCTTCATAGCAACTGATCC |  |  |  |  |  |  |  |  |
| PKM1 | F | ACCGCAAGCTGTTTGAAGAA | 58 | NM_182471.4 | [3](https://doi.org/10.1371/journal.pone.0057610) |  |  |  |  |  |
|  | R | TCCATGAGGTCTGTGGAGTG |  |  |  |  |  |  |  |  |
| GSK3B | F | AGACGCTCCCTGTGATTTATGT | 89 | NM_002093.4 |  |  |  |  |  |  |
|  | R | CCGATGGCAGATTCCAAAGG |  |  |  |  |  |  |  |  |
| PDHA1 | F | TGGTAGCATCCCGTAATTTTGC | 151 | NM_000284.4 |  |  |  |  |  |  |
|  | R | ATTCGGCGTACAGTCTGCATC |  |  |  |  |  |  |  |  |
| G6PD | F | CGAGGCCGTCACCAAGAAC | 166 | NM_000402.4 |  |  |  |  |  |  |
|  | R | GTAGTGGTCGATGCGGTAGA |  |  |  |  |  |  |  |  |
| HK1 | F | GCTCTCCGATGAAACTCTCATAG | 121 | NM_033498.3 |  |  |  |  |  |  |
|  | R | GGACCTTACGAATGTTGGCAA |  |  |  |  |  |  |  |  |
| GFAP | F | TATGAGGCAATGGCGTCCAG | 130 | NM_002055.5 |  |  |  |  |  |  |
|  | R | AGTCGTTGGCTTCGTGCTTG |  |  |  |  |  |  |  |  |
| SLC2A1 | F | GGCCAAGAGTGTGCTAAAGAA | 201 | NM_006516.4 |  |  |  |  |  |  |
|  | R | ACAGCGTTGATGCCAGACAG |  |  |  |  |  |  |  |  |
| IDE | F | TTTTCAGCCCATTTGCTTATGTG | 92 | NM_001165946.2 |  |  |  |  |  |  |
|  | R | TGCATACTCGTTGAGTGAGTCTT |  |  |  |  |  |  |  |  |
| TNF | F | ATGGGCTACAGGCTTGTCACTC | 135 | NM_000594.4 | 4 |  |  |  |  |  |
|  | R | CTCTTCTGCCTGCTGCACTTTG |  |  |  |  |  |  |  |  |
| 1L1B | F | ATGATGGCTTATTACAGTGGCAA | 132 | NM_000576.3 |  |  |  |  |  |  |
|  | R | GTCGGAGATTCGTAGCTGGA |  |  |  |  |  |  |  |  |
| INSR | F | AAAACGAGGCCCGAAGATTTC | 90 | NM_000208.4 |  |  |  |  |  |  |
|  | R | GAGCCCATAGACCCGGAAG |  |  |  |  |  |  |  |  |
| LEPR | F | ACCTCTGGTTCCCCAAAAAGG | 89 | NM_002303.6 |  |  |  |  |  |  |
|  | R | TTGGCACAGGCACAAGACAT |  |  |  |  |  |  |  |  |
| IGF1R | F | TACTTGCTGCTGTTCCGAGTGG | 101 | NM_000875.5 |  |  |  |  |  |  |
|  | R | AGGGCGTAGTTGTAGAAGAGTTTCC |  |  |  |  |  |  |  |  |
| PTPN1 | F | CGACCAGCTGCGCTTCTC | 61 | NM_002827.4 |  |  |  |  |  |  |
|  | R | GTCCCCCATGATGAATTTGG |  |  |  |  |  |  |  |  |
| PTPN11 | F | CCCACATCAAGATTCAGAACACT | 105 | NM_002834.5 |  |  |  |  |  |  |
|  | R | GCCCGTGATGTTCCATGTAA |  |  |  |  |  |  |  |  |
| NDUFC1 | F | AGTGCGATCAAAGTTCTACGTG | 89 | NM_001184986.1 | 5 |  |  |  |  |  |
|  | R | AGAAGACAGTGGTGCCCAAG |  |  |  |  |  |  |  |  |
| AIF1 | F | GACCTTAATGGAAATGGCGATA | 172 | NM_032955.3 |  |  |  |  |  |  |
|  | R | ATCTCTTGCCCAGCATCATC |  |  |  |  |  |  |  |  |
|  |  |  |  |  |  |  |  |  |  |  |
| Gene |  | Sequence (5'-3') | Product (bp) | Accession number | Reference |  |  |  |  |  |
| BACE1 | F | ACCAACCTTCGTTTGCCCAA | 101 | NM_012104.6 |  |  |  |  |  |  |
|  | R | TCTCCTAGCCAGAAACCATCAG |  |  |  |  |  |  |  |  |
|  |  |  |  |  |  |  |  |  |  |  |

|  | Reference |  |
| --- | --- | --- |
|  | 1 | <https://doi.org/10.1038/srep37116> |
|  | 2 | <https://doi.org/10.1038/cddis.2014.292> |
|  | 3 | https://doi.org/10.1371/journal.pone.0057610 |
|  | 4 | <https://doi.org/10.1186/s12929-017-0383-3> |
|  | 5 | https://doi.org/10.3389/fonc.2021.709044 |
