# Supplementary figures and images for: Dysregulated glucose metabolism in the visual cortex of human subjects with mild cognitive impairment and Alzheimer’s disease
Source: Front Aging Neurosci. 2026 Apr 9;18:1710075. doi: 10.3389/fnagi.2026.1710075 (PMC13102864; doi:10.3389/fnagi.2026.1710075)

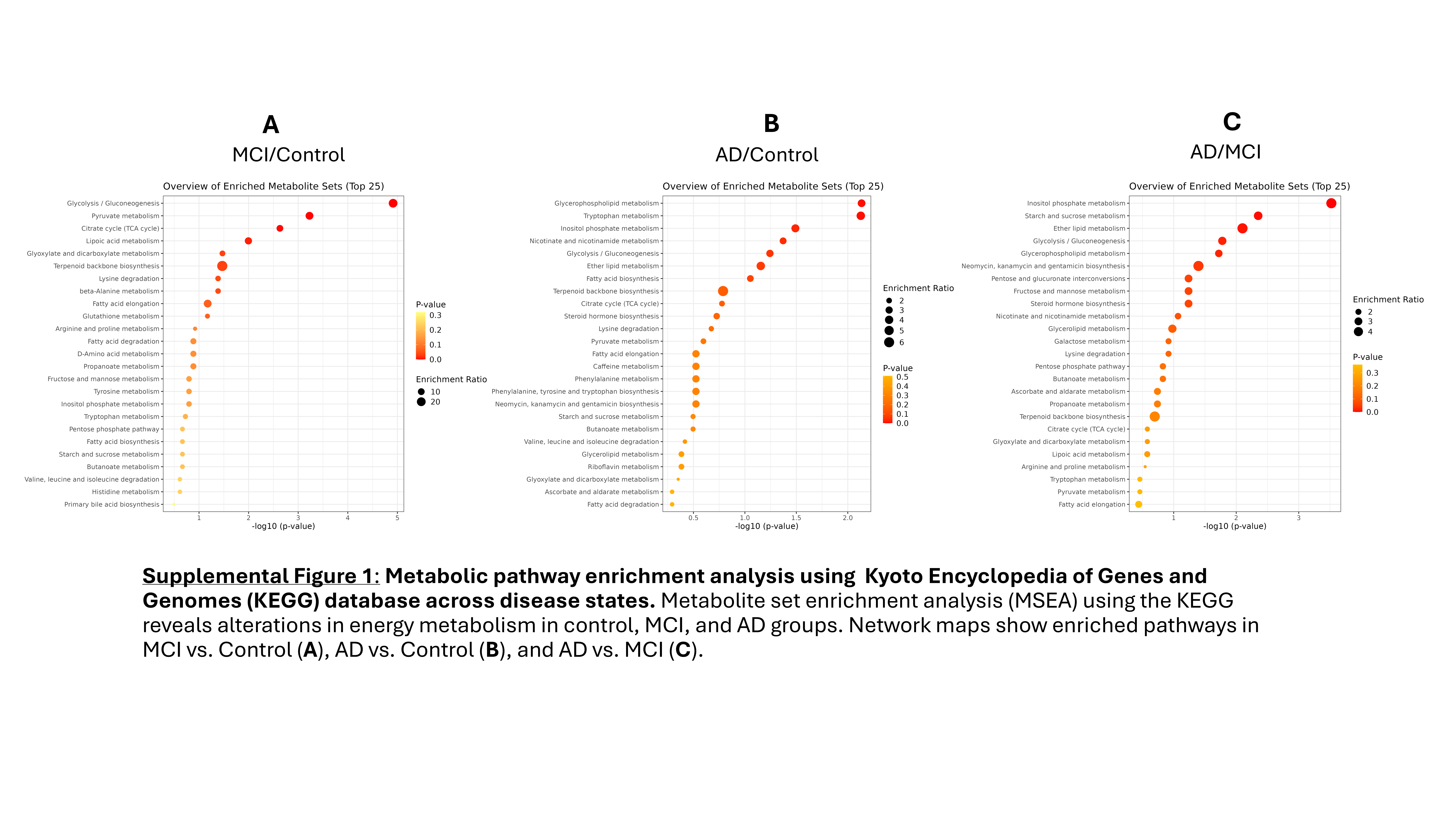

Supplement: Supplementary file 6 [file Image_1.tif]

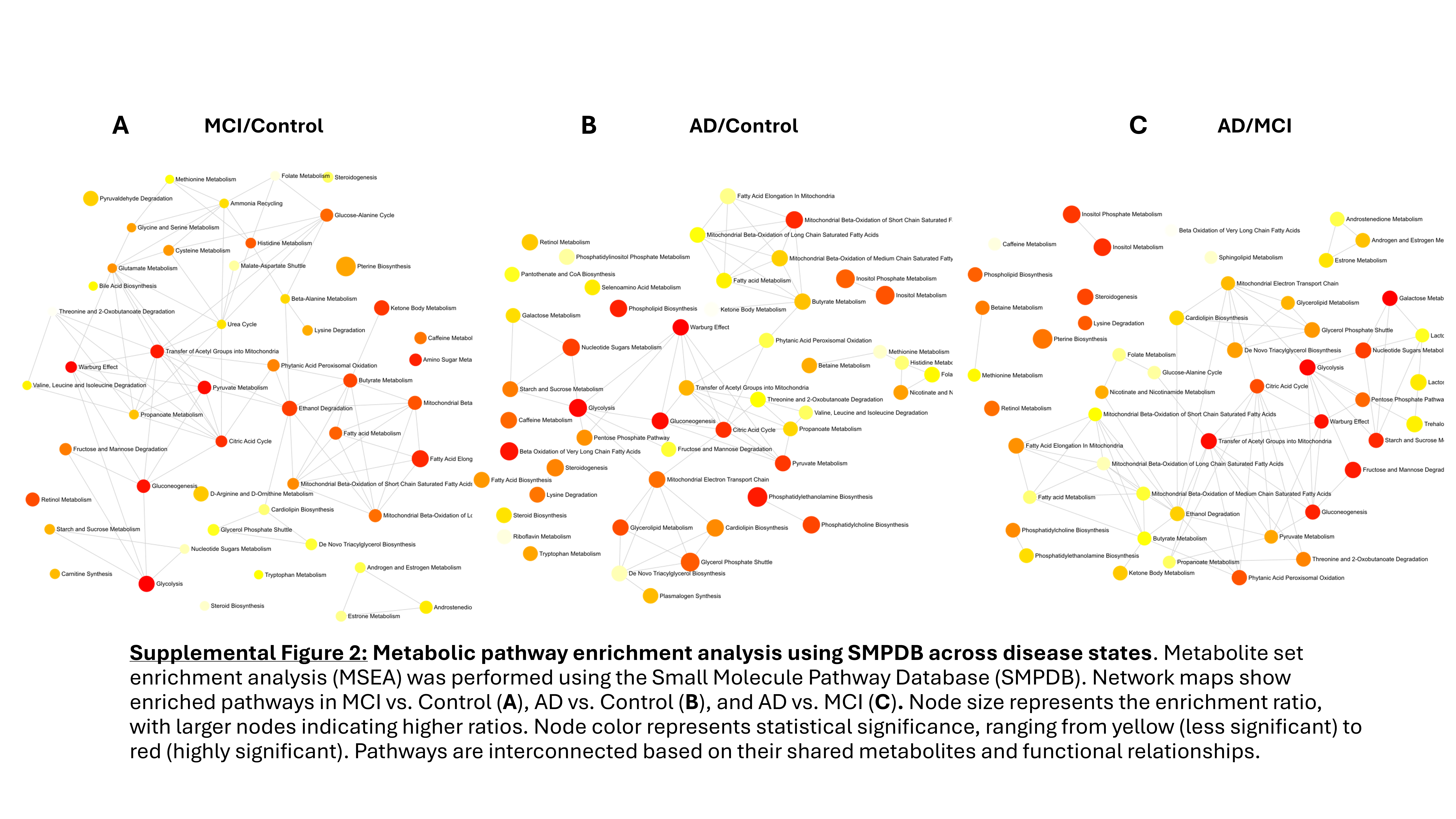

Supplement: Supplementary file 7 [file Image_2.tif]

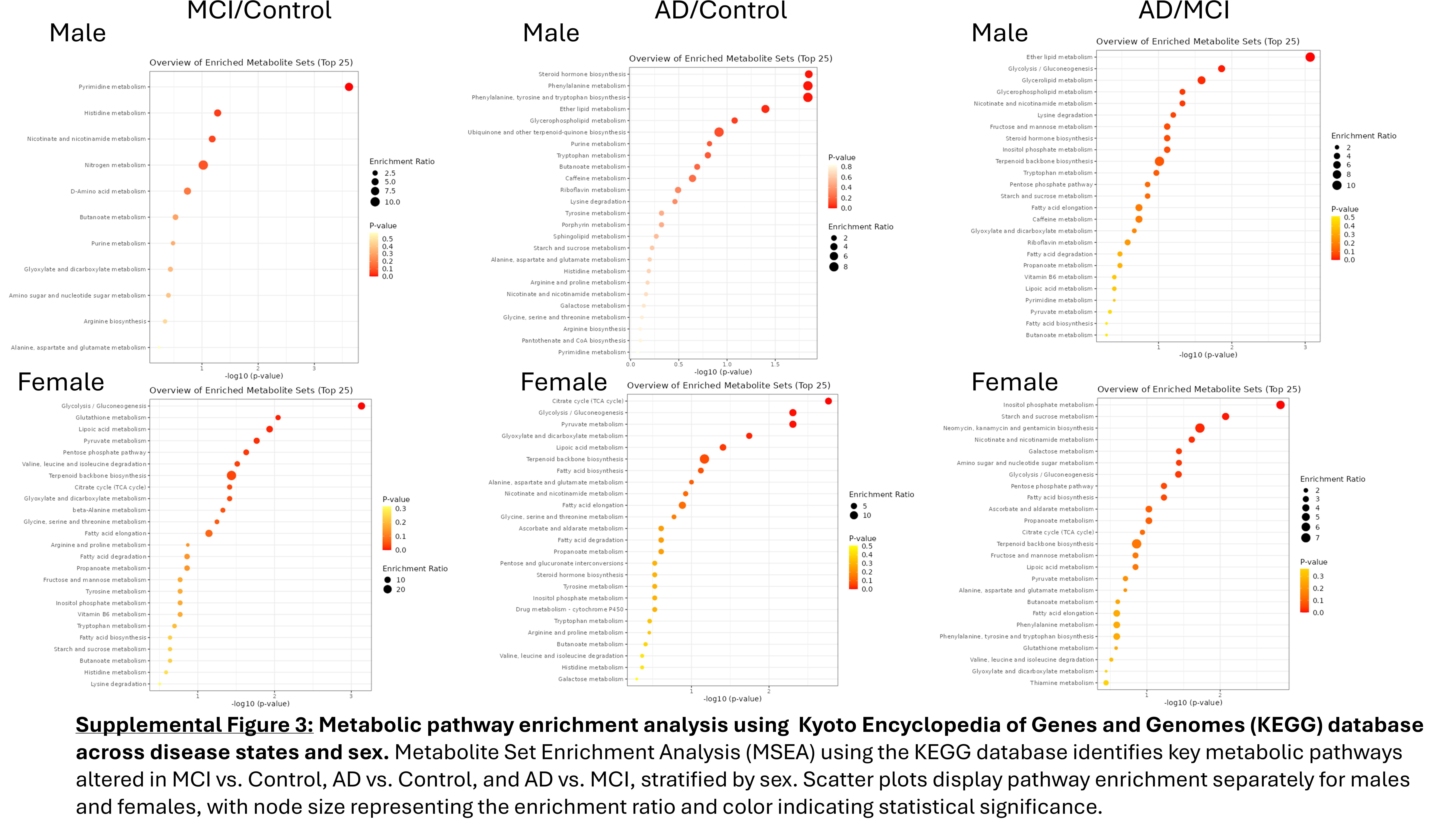

Supplement: Supplementary file 8 [file Image_3.tif]

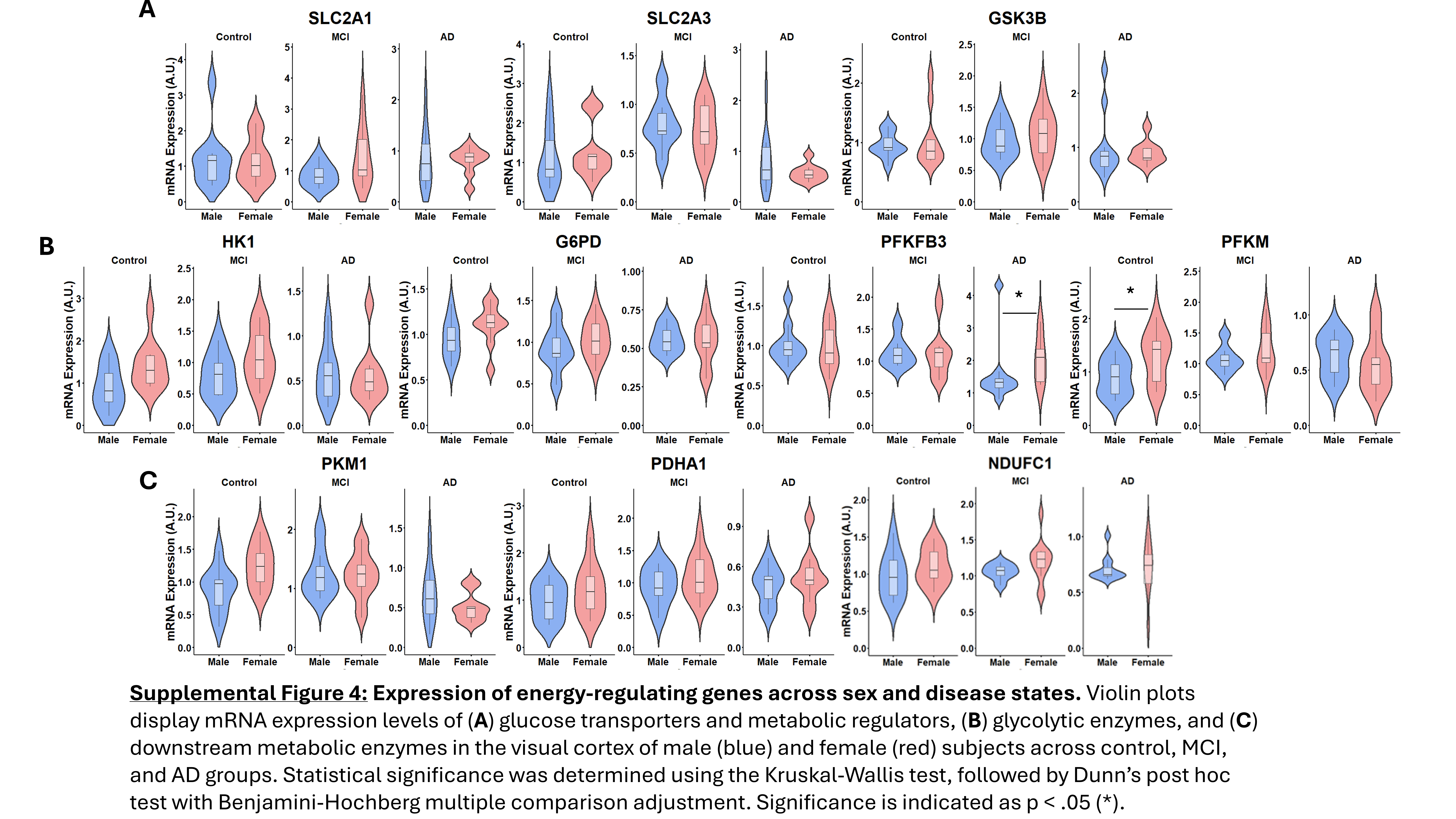

Supplement: Supplementary file 9 [file Image_4.tif]

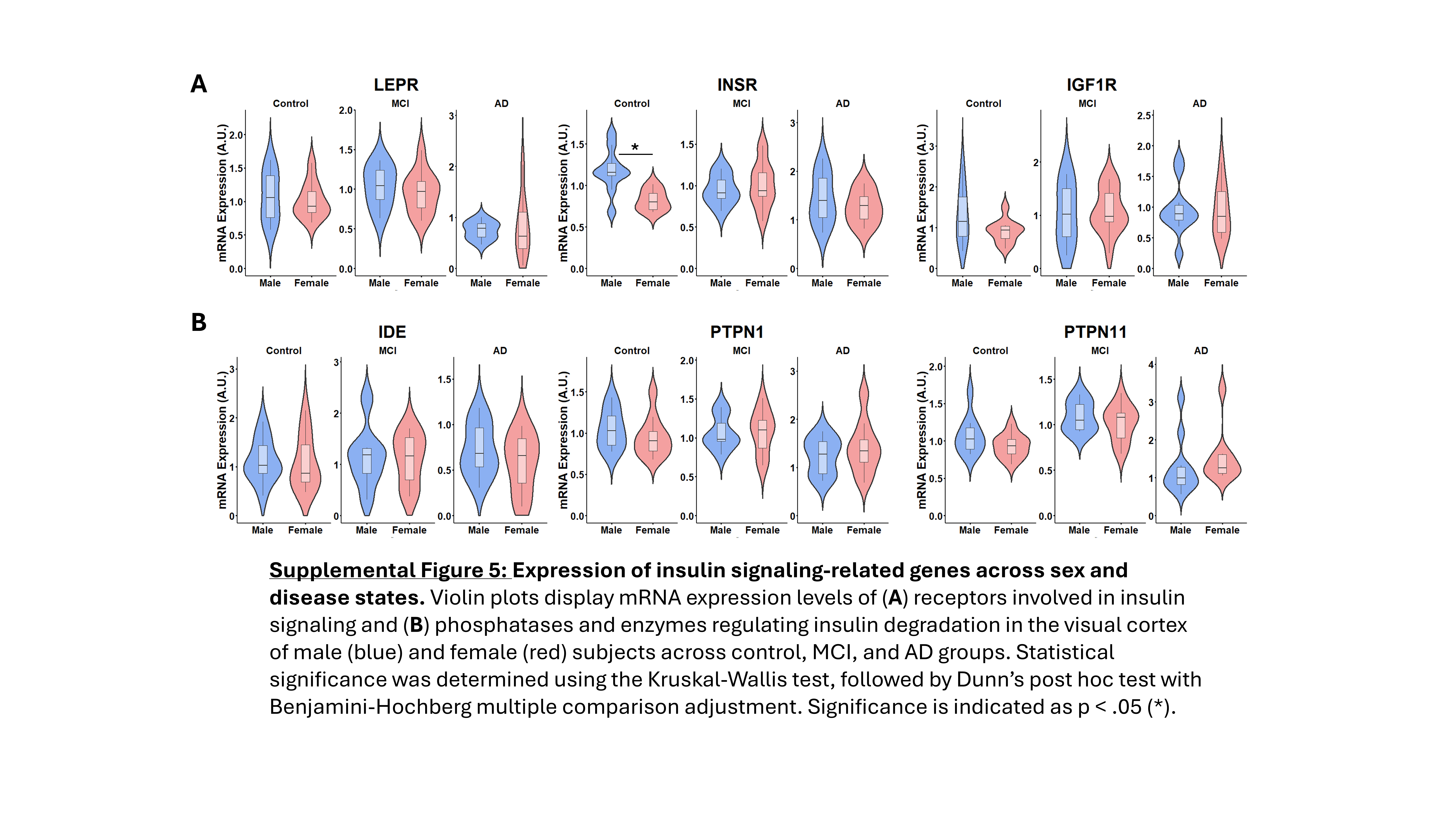

Supplement: Supplementary file 10 [file Image_5.tif]

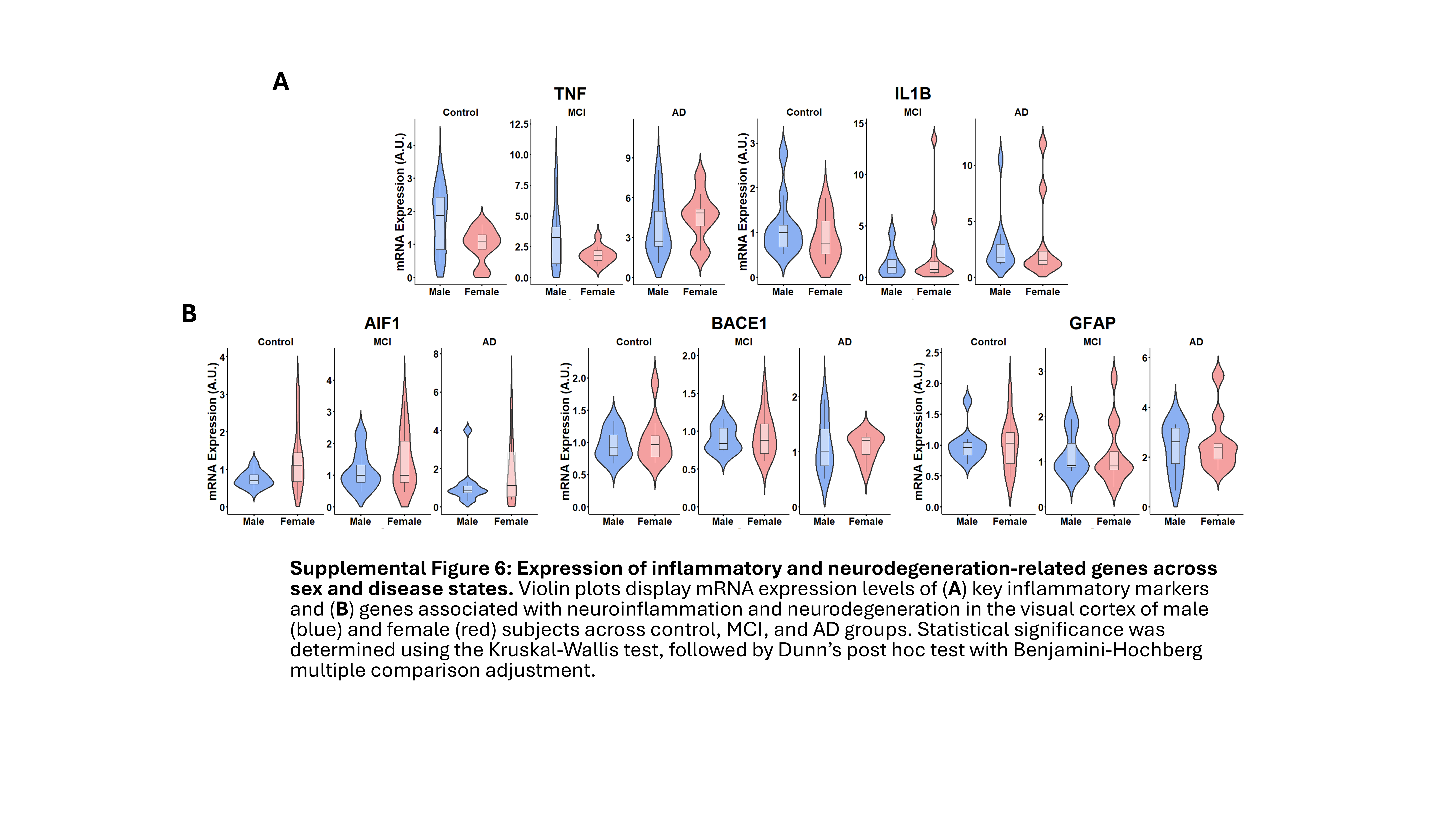

Supplement: Supplementary file 11 [file Image_6.tif]

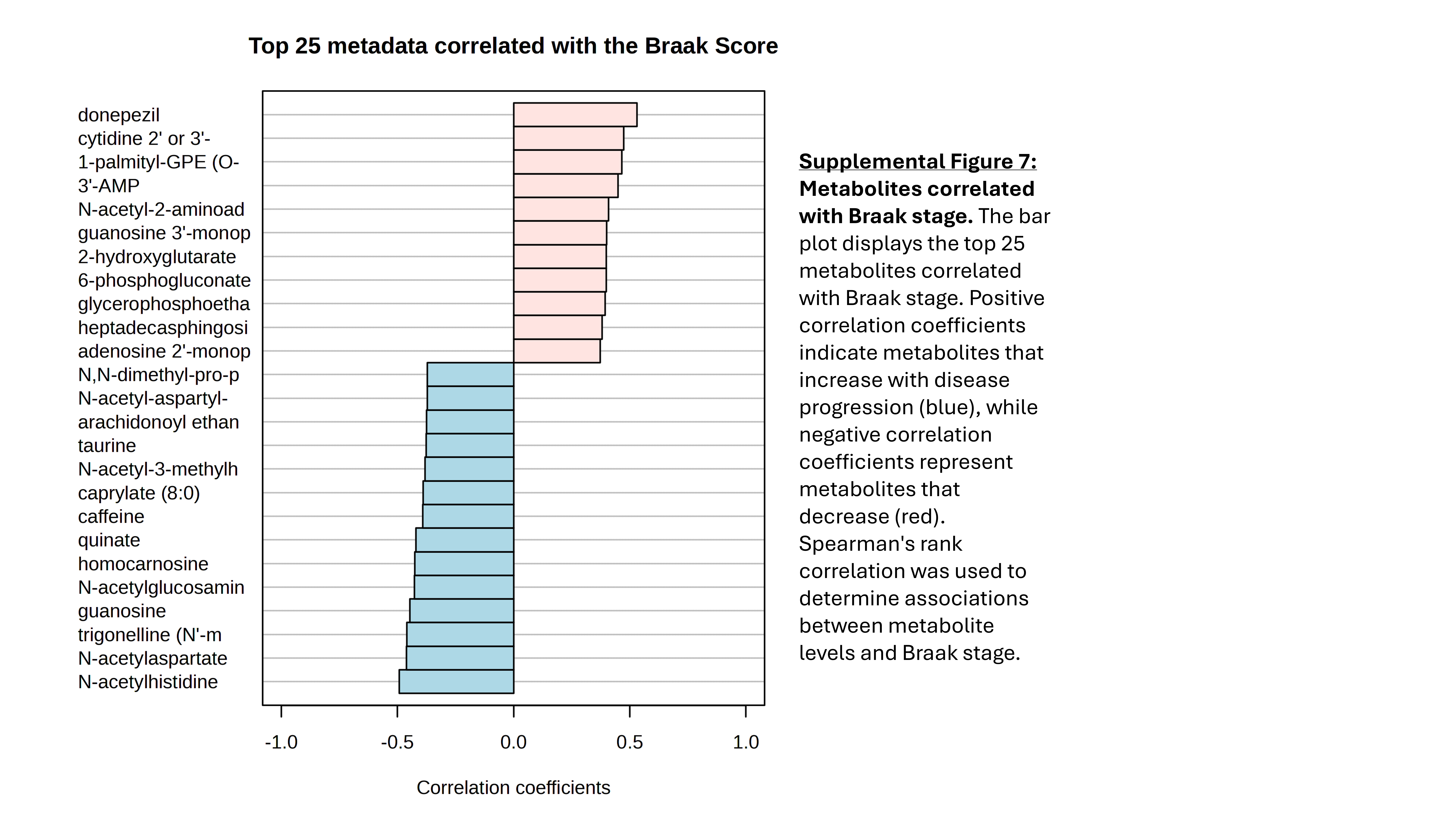

Supplement: Supplementary file 12 [file Image_7.tif]
